# Experimental assessment of biotic and abiotic filters driving community composition

**DOI:** 10.1002/ece3.6461

**Published:** 2020-06-13

**Authors:** Eva Švamberková, Jan Lepš

**Affiliations:** ^1^ Department of Botany Faculty of Science University of South Bohemia České Budějovice Czech Republic; ^2^ Institute of Entomology Biology Centre Czech Academy of Sciences České Budějovice Czech Republic

**Keywords:** abiotic filter, Beals index, biotic filter, competitive exclusion, sowing and transplanting experiment, species pool

## Abstract

Species occurrence in a site can be limited by both the abiotic environment and biotic interactions. These two factors operate in concert, but their relative importance is often unclear. By experimentally introducing seeds or plants into competition‐free gaps or into the intact vegetation, we can disentangle the biotic and abiotic effects on plant establishment.

We established a seed‐sowing/transplant experiment in three different meadows. Species were introduced, as seeds and pregrown transplants, into competition‐free gaps and the intact vegetation. They included 12 resident plants from the locality and 18 species typical for different habitats. Last two years, gaps were overgrown with vegetation from surrounding plants and we observed the competitive exclusion of our focal plants. We compared plant survival with the expected occurrence in target locality (Beals index).

Many of the species with habitat preferences different from our localities were able to successfully establish from seeds and grow in the focal habitat if competition was removed. They included species typical for much drier conditions. These species were thus not limited by the abiotic conditions, but by competition. Pregrown transplants were less sensitive to competition, when compared to seedlings germinated from seeds. Beals index significantly predicted both species success in gaps and the ability to withstand competition. Survival in a community is dependent on the adaptation to both the abiotic environment and biotic interactions. Statistically significant correlation coefficients of the ratio of seedling survival in vegetation and gaps with Beals index suggest the importance of biotic interactions as a determinant of plant community composition.

To disentangle the importance of abiotic and biotic effect on plant establishment, it is important to distinguish between species pool as a set of species typically found in given community type (determined by Beals index) and a set of species for which the abiotic conditions are suitable.

## Graphical abstract

We demonstrated in a six‐year experiment that a main driver of species community composition is a biotic filter because many species with habitat preferences different from focal localities (i.e. with low Beals index) were able to successfully establish from seeds and grow in the focal habitat if competition was removed. Some of these species that became established in gaps were able to exclude or at least postpone their competitive exclusion. Although Beals index is a good predictor of species survival in plant communities, we should be careful to use it as species pool determinant, especially in disentangling the effect of abiotic and biotic filter on species community composition because it can exclude many species able to grow in given abiotic environment from species pool.

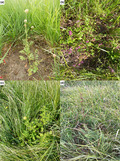

## 1 INTRODUCTION

Each plant community is formed by a subset of the species pool, that is, a subset of all species available to colonize a given site (Cornell & Harrison, 2014). The basic question is then which mechanisms decide which species from the species pool will finally form the community.

Dispersal limitation is an important factor for species occurring in the region. For example, the successful establishment of a single individual often requires the arrival of hundreds or thousands of seeds (Vítová & Lepš, 2011). Interestingly, low favorability of a particular habitat can be overcome by massive numbers of propagules (Fibich, Vítová, & Lepš, 2018). Nevertheless, the main processes limiting species occurrence in a local scale are abiotic environment and biotic interactions (HilleRisLambers, Adler, Harpole, Levine, & Mayfield, 2012). Abiotic environment is influenced by many factors such as temperature and precipitations, availability of nutrients and other resources which plants need for their survival. Biotic interactions include the relationships among living organisms in a community. Although other biotic interactions (e.g., mycorrhiza, facilitation, pollination, herbivory) play an important role in plant communities, competition is considered a significant factor that limits co‐occurrence among species (Grubb, 1977; Li, Poisot, Waller, & Baiser, 2018; Palmer, 1994; Wellstein et al., 2014). Furthermore, studies typically use competition as biotic filter in community assembly studies (HilleRisLambers et al., 2012).

In local communities, abiotic environment and biotic interactions operate simultaneously, but their relative importance in structuring local community composition is often unknown and difficult to disentangle on the basis of observational data only (Araújo & Rozenfeld, 2014; Cadotte & Tucker, 2017; Kraft et al., 2015). Although many studies based on observational data use the concept of environmental filtering as the effect of abiotic environment only, they in fact reflect environmental filtering which includes not only the species ability to survive under specific environmental condition of the given site but also withstand under the competition of other species present in a given site (Cadotte & Tucker, 2017). By this approach, the effect of biotic interactions on local community structuring could be significantly underestimated. Very probably, only experimental approach manipulating biotic interactions in species communities can reliably distinguish the effect of abiotic environment and biotic interactions (Kraft et al., 2015). Nevertheless, some studies (e.g., D'Amen, Mod, Gotelli, & Guisan, 2018) claim that the analysis based on combination of observational data and null models is able to separate the effect of biotic filter from the environmental filtering.

Sowing and transplant experiments are excellent approaches to disentangle the effects of various “filters” on community composition (Švamberková, Vítová, & Lepš, 2017; Turnbull, Crawley, & Rees, 2000; Zobel & Kalamees, 2005). Excluding dispersal limitation, failure to establish after sowing or transplanting can be attributed to habitat limitation. There are many examples of species that are able to grow in given abiotic conditions, but are excluded by the biotic filter. These species are present within a regional species pool, but are representative for very different habitats. In order to examine the ability of these species to withstand the abiotic conditions of a given habitat, seed/transplant introduction experiments, where biotic filters (especially competition) are experimentally removed, are required (Cornell & Harrison, 2014; Švamberková et al., 2017). Species that successfully establish in competition‐free experimental plots should be considered a part of the species pool defined as species able to pass only through abiotic filters (Butaye, Jacquemyn, Honnay, & Hermy, 2001) while they cannot be a part of usually used species pool defined as species able to pass through the both abiotic and biotic filters (Zobel, 1997). Comparing plant performance across artificial competition‐free gaps and intact vegetation (where the biotic and abiotic filters work in concert) can separate the importance of biotic and abiotic effects on plant establishment (HilleRisLambers et al., 2012; Kraft et al., 2015).

Many species require some type of gap (i.e., plot with reduced competition) in natural settings (Puerta‐Piñero, Muller‐Landau, Calderón, & Wright, 2013). In nature, gaps are the result of various disturbances, which create competition‐free microhabitats and enable species to germinate and subsequently establish. When studying species establishment in seed/transplant introduction experiments, competition can be artificially excluded (or substantially reduced) using experimentally generated gaps (Kotorová & Lepš, 1999; Lemke, Janßen, & Porembski, 2015; Tofts & Silvertown, 2002; Vítová, Macek, & Lepš, 2017). In gaps, competition for light, nutrients, and water is reduced (Frei, Scheepens, & Stöcklin, 2012; Lemke et al., 2015). On the other hand, species present in gaps are more exposed to extreme environmental conditions, such as desiccation (Kotorová & Lepš, 1999; Vítová & Lepš, 2011). Seedlings growing in gaps are also more apparent to herbivores than seedlings occurring within intact vegetation (Gustafsson, Ehrlén, & Eriksson, 2002; Lemke et al., 2015). Both gap size and the time of their formation play a crucial role in the establishment of new seedling species, affecting which species is first to colonize this gap. Even so, the establishment of seedlings in a community is unlikely and seedling survival does not always assure the long‐term persistence of the species (Gustafsson et al., 2002; Vítová & Lepš, 2011; Zobel, 1997).

Most species are filtered out of a community during the germination phase and subsequent establishment of individuals (Kotorová & Lepš, 1999). The importance of factors (both abiotic and biotic) affecting species survival in a community can differ in different life stages of plants because their regeneration and realized niches are often quite distinct (Grubb, 1977). One of the primary reasons for the absence of some species in a community is their inability to establish in the presence of competition from other species. Although biotic interactions affect plants in later stages of their life span, the effect is not as strong as in their early phases of seedling development because older individuals are more biotic resistant than small seedlings (Bennett et al., 2016; Tofts & Silvertown, 2002). It suggests that competitive exclusion of well‐established individuals in a community may be rather slow (Adler, Ellner, & Levine, 2010). The studying of different life stages is thus necessary to get a complete insight into local processes influencing a whole life cycle of species.

When comparing the effect of abiotic and biotic filter on species composition of a local community, we need to define a local species pool, ideally as the ability of a given species to establish based on the abiotic environment alone without the effect of competition filter (Butaye et al., 2001; Švamberková et al., 2017). There are various methods to help determine the species pool: Ellenberg indicator values (Pärtel, Zobel, Zobel, van der Maarel, & Partel, 1996; Zobel, 1997; Zobel, van der Maarel, & Dupré, 1998), functional traits (de Bello et al., 2012; Moor, Hylander, & Norberg, 2015; Sonnier, Shipley, & Navas, 2010), phytosociological knowledge from local experts (Sádlo, Chytrý, & Pyšek, 2007), Beals index (Botta‐Dukát, 2012; Ewald, 2002; Münzbergová & Herben, 2004), or ordination methods (Brown et al., 2019). Nevertheless, with exception of experimental approach, all other methods of species pool determination reflect the influence of the both biotic and abiotic filters. Nevertheless, because experimental approach is very time consuming, Beals index can be quite invaluable approach to species pool assessment. While most of the above‐mentioned approaches for determination of species pool size depend on either expert's phytosociological experience or models corresponding with environmental gradients, methods related to Beals index employ information based on multivariate structure of real data. It compares species co‐occurrence of examined species with other species of the appropriate habitat from a database of many phytosociological relevés (Chytrý & Rafajová, 2003), reflecting thus concerted effect of biotic and abiotic filters. Although Beals index is, in fact, also one of the phytosociological methods, neither any classification nor any environmental gradients determined in advance are employed. It transforms a species pool definition from a strictly determined set of species into species occurrence probability (Botta‐Dukát, 2012).

We conducted a seed/transplant introduction experiment across three different meadow habitats (Appendix S1). Species, both resident in the locality and typical for different habitats (not expected to be part of the species pool), were introduced as either seeds or pregrown transplants into either competition‐free gaps or the intact vegetation. Subsequently, we computed the expected occurrence of species from our experiment on target habitats using Beals index derived from the species co‐occurrence pattern in the National Phytosociological Database (Chytrý & Rafajová, 2003) and compared these results with the real plant survival from our experiment. During the last two years of the experiment, surrounding vegetation was left to overgrow into gaps and we observed the competitive exclusion of our focal plants.

Our study aimed to (a) compare the species pool determined by seed/transplant introduction experiment with the species pool delimited using Beals index; (b) disentangle the importance of the biotic and abiotic effects on plant establishment via the removal of competition; and (c) compare the survival of target species in different life stages (i.e., sown as seeds and planted as pregrown transplants) and their competitive exclusion.

We expect that (a) some species determined by Beals index as improbable to occur in target habitats will be able to establish experimentally in competition‐free gaps. (b) Both abiotic and biotic effects will influence the species establishment, but competition will be the most important determinant. We suggest that if survival is affected by both intrinsic characteristics of individual species and their interaction with the environment, the more an environment discriminates among species, correlations of species successes across ecologically different habitats should be weaker. In this way, we can identify, whether the discrimination among species is more pronounced in gaps (suggesting mainly effect of abiotic environment), or in controls (discrimination by the whole habitat including competition by extant vegetation). (c) Competitive exclusion will be more important for seedlings growing from seeds in the field than for pregrown transplants.

## 2 MATERIALS AND METHODS

### 2.1 Study site

The experiment was conducted in the northeastern region of Czech Republic, in a species‐rich locality named Strašovský rybník (50°6'N, 15°31'E, 217 m a.s.l.). The study site contained a pond, surrounded by a mosaic of wet meadows and fens. A littoral zone of the pond, with stands of *Phragmites australis*, accounted for the largest area. These reed beds are bordered by stands of tall sedges, with the remaining part of the locality being composed of *Molinion* and *Arrhenatherion* meadows with small patches of alluvial meadows and calcareous fens. The climatic conditions during the years of our experiment are provided in Table S1.

Our experiment was carried out in locations (at least 200 m distant from each other), which were referred to according to their two main dominant plants: (a) “*Carex acuta‐Carex panicea*” (50°6'0.8"N, 15°31'0.5"E), (b) “*Deschampsia caespitosa‐Carex tomentosa*” (50°5'59.4"N, 15°31'11.3"E), and (c) “*Sesleria uliginosa‐Briza media*” (50°5'57.6"N, 15°31'14.4"E) habitats, respectively. Moisture regime of all three habitats was dynamic in time (Figure S1, Table S4) and contained distinct species compositions (Figure S2). They differed in overall productivity (Tables S2 and S4) and several soil characteristics (Table S3). Between 2013 (i.e., the first year of our experiment) and 2016, all three habitats, as well as our experimental plots, were mowed regularly twice a year at the end of June and in mid‐October; with the exception in 2015 when only one mowing event occurred due to an abnormally dry summer. Since 2017, the study locations, including our plots, were mowed only once a year.

### 2.2 Seed introduction experiment

To assess species establishment and survival in the presence and absence of competition, we introduced seeds and pregrown young individuals (transplants) of both resident and nonresident plant species to our three habitats (Appendix S1). We selected species with good germination rate (knowledge from previous studies, e.g., Švamberková et al., 2017) from species typical for the region of our target locality. A species residence was determined for individual habitats based on whether a species was present in at least one of the five phytosociological relevés (5 × 5 m) of given habitat type recorded in June 2014 (i.e., “habitat residency,” Table S5). We also used an additional classification, where any species present in at least one habitat type (according to phytosociological relevés from June 2014) or found within the study site during the nature conservation‐screening inventory by Jan Horník et al. (unpublished data) were considered residents for the entire locality (i.e., “whole locality residence,” Table S5). Nonresident species include species typical for both drier and wetter conditions than target locality. Nevertheless, all the nonresident species can be considered part of the regional species pool, because they are found in close surrounding (see maps of species distribution at www.pladias.cz/en/, accessed on May 8, 2019) and their propagules are thus able to reach the target locality. Seeds and transplants were placed into either control plots, with the intact vegetation, or artificially created gaps.

We created 30 artificial gaps (40 × 40 cm) in two replications in each habitat type, each by digging a hole 20 cm deep, and refilling with soil from the target habitat. To prevent competition from surrounding vegetation, gaps were weeded regularly two times a year (in spring and autumn) until 2016 when gaps were weeded once during spring for the last time. In 2017 and 2018, we observed the potential competitive exclusion of established individuals in gaps from the neighboring vegetation. Control plots of the same size were established without any manipulation of extant vegetation. Seeds from 30 species, 12 residents and 18 nonresidents (Table S5), were sowed to the center of 20 × 20 cm plots within gap and control treatments in spring 2013. We used seeds from a commercial supplier (Planta Naturalis, Markvartice, Czech Republic). Each species was sowed separately in its own plot. Within a plot, 200 seeds of species, which had a seed weight of one seed 1 mg or more, were sown for each plant species. We sowed more than 200 seeds for plant species with seeds lighter than 1 mg because small seeds are expected to have reduced probability of establishment (Cornelissen et al., 2003). We used an ad hoc formula to increase the amount of seeds lighter than 1 mg: *x* = 200 (1 − log *m*), where *x* was a weight of seeds required for sowing and *m* a weight of one seed in mg. This process helped provide enough individuals for the assessment of mortality. The success of seedling establishment and survival was subsequently expressed as the number of survivors out of the number of the sown seeds. The proportion of seedling recruitment and survival was monitored from 2013 to 2018 several times per year.

### 2.3 Transplant experiment

Transplants of the same species used in the seed introduction experiment (Table S6) were pregrown in jiffy peat pots in a growth chamber (12 hr light and 12 hr darkness, 19°C) during 50 days. These transplants were planted within a 10‐cm wide border region of the same gap and control plots as those used for the seed introduction experiment. We completely excluded six species from the transplant experiment (i.e., from all habitat types) and four others only from *Carex acuta‐Carex panicea* habitat and from one replication of *Deschampsia caespitosa‐Carex tomentosa* habitat because their pregrowth was unsuccessful (Table S6). In all other cases, three transplants of each species were planted and their initial height and number of leaves were measured (Table S6). All transplants were planted in target habitats at the end of May 2013, with the exception of the *Carex acuta‐Carex panicea* habitat, where they were transplanted in the second half of June because of an unexpected flood. Transplant survival was monitored from 2013 to 2018 several times per year and subsequently compared with success of seedlings in the seed introduction experiment.

### 2.4 Data analysis

We used the ratio of the living individuals, to the number of seeds sown/planted transplants as our measurement of success for individual species. This measurement was characterized for each sampling date and combination of habitat and treatment (i.e., gap/control). Each value is represented as the average of two replications. For convenience, we use the term *survival* throughout the text, but acknowledge that it is the outcome of germination (in case of sown seeds) and establishment success and survival.

Seedling and transplant survival were analyzed using a repeated measures (split‐plot) ANOVA in Statistica 13 (StatSoft, 2015), where time and treatment were modeled as within subject effects and species residence as a between subject effect. This analysis was carried out for each habitat separately. In a subsequent analysis, habitat type, time, and treatment were modeled as within subject effects and species residence as a between subject effect. Species identity was not included in these analyses. Prior to both analyses, survival of seedlings and transplants were arcsine transformed to help meet assumptions for ANOVA.

For each sown species and habitat, we calculated a Beals index (Beals, 1984) as an average of the conditional probability of a focal species occurrence, provided the presence of the other species in the target habitat relevé (five 5 × 5 m relevés per habitats were recorded in June 2014): $P_{\mathrm{ij}}=\frac{1}{S_{i}}\sum_{k\neq j}\frac{N_{\mathrm{jk}}}{N_{k}}$ where *P_ij_* is the estimate of probability to find species *j* in habitat *i* (i.e., the Beals index), *S_i_* is the number of species in a relevé characterized by habitat *i* (minus 1 if species *j* is present), *N_jk_* is the number of joint occurrences of species *j* and *k*, and *N_k_* is number of occurrences of species *k* in the reference database, where *k* is index of species in the relevé (Münzbergová & Herben, 2004). The Czech National Phytosociological Database (Chytrý & Rafajová, 2003) in stratified form to reduce oversampling of some areas (Těšitel, Fibich, de Bello, Chytrý, & Lepš, 2015) was used as the reference database. After the subsampling, the reference database contained 31,512 relevés. We used the weighted form of the Beals index, that is, the function “beals” of “type” = 2 (abundances were used to compute weighted averages of conditioned probabilities instead of the plain average used in the above formula) in the R‐package “vegan” (Oksanen et al., 2019). The index was calculated for each relevé separately, and the average value across the five phytosociological relevés per each habitat was subsequently used. Beals index can be thus considered a measure of favorability of habitat for a given species.

For each combination of observation time, habitat, and species, we calculated average survival (from two replications) in gaps and controls, and the ratio of average control/gap survival. This ratio provided an estimate of competitive reduction, where a value of 1 denotes no effect of competition and 0 signifies the strongest effect of competition. In cases where survival in gaps was zero, the effect of competition could not be estimated and thus was not considered in subsequent analyses performed in Statistica 13 (StatSoft, 2015). We tested for significant correlations between Beals index and species survival across the different treatments to examine whether we can predict habitat favorability for a species. We also calculated the correlation of species survival always between two different habitats (each habitat taken in pair with each other habitat), for gaps and controls separately to identify, whether the differentiation in species survival between two habitats is determined mainly by abiotic environment or biotic interactions. Higher correlation coefficients for species survival in gaps than for control plots mean the more important discrimination of species between these two habitats by the biotic interactions than by abiotic environment.

## 3 RESULTS

### 3.1 Seed germination and survival of seedlings in contrast to transplants

Most of the 30 sown species succeeded in germination in target habitats (the highest germination success averaged over the three habitats was 42% in a gap for *Plantago lanceolata*, and 12% in intact vegetation for *Nardus stricta*, median was 5.43% in gap and 0.62 in intact vegetation). Only two of the 18 nonresident species (*Bistorta major* and *Viola hirta*) did not successfully germinate in any habitat type. *Lathyrus vernus* was unable to germinate in the *Carex acuta‐Carex panicea* habitat, but it was able to germinate in the other two habitats, but only in gaps. *Bupleurum falcatum* successfully germinated in *Carex acuta‐Carex panicea* and *Deschampsia caespitosa‐Carex tomentosa* habitat gaps, but it was unable to germinate in *Sesleria uliginosa‐Briza media* habitat. All 12 sown resident species germinated in all habitat types.

**FIGURE 1:**
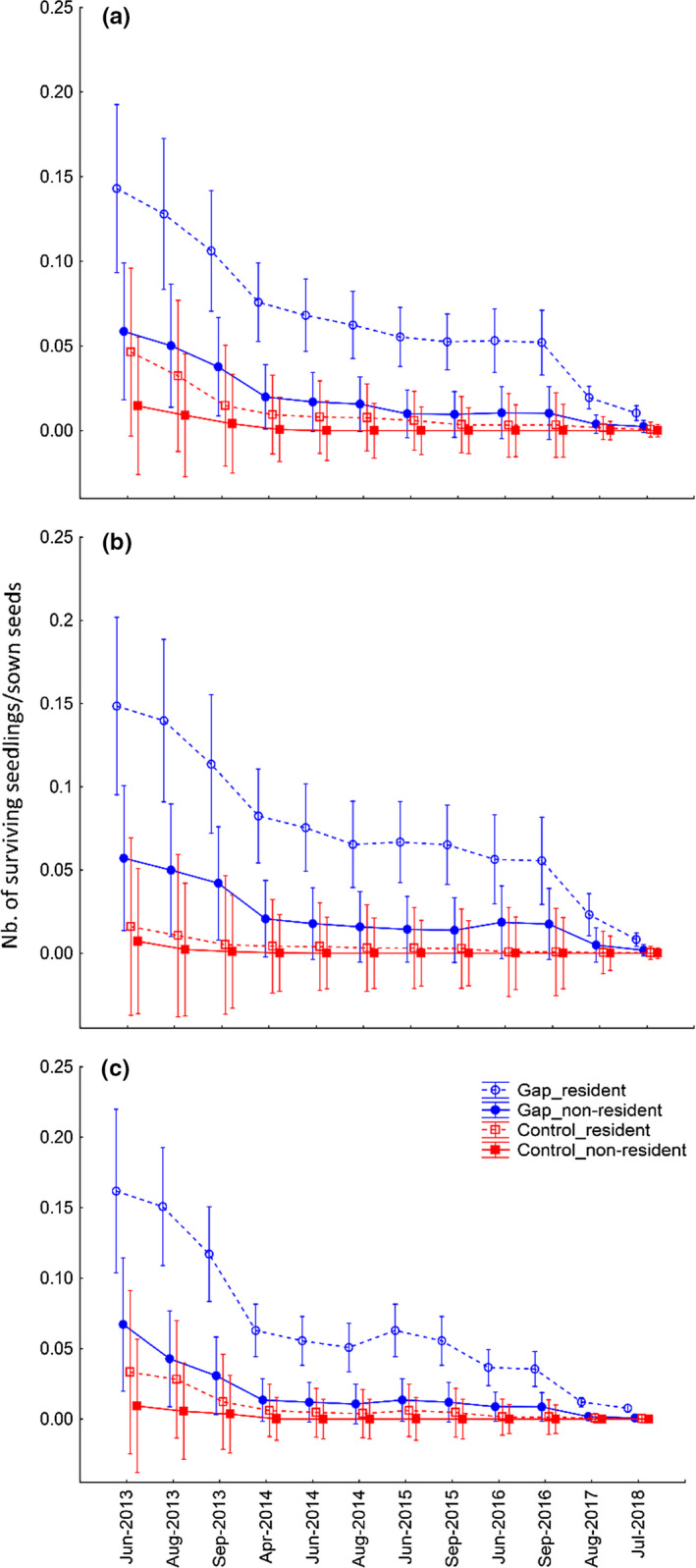
Average survival of resident and nonresident seedlings in gaps and intact vegetation (control) during the experiment within each habitat: (a) *Carex acuta‐Carex panicea*, (b) *Deschmpsia caespitosa‐Carex tomentosa*, and (c) *Sesleria uliginosa‐Briza media* habitat. Error bars represent 95% confidence intervals

**FIGURE 2:**
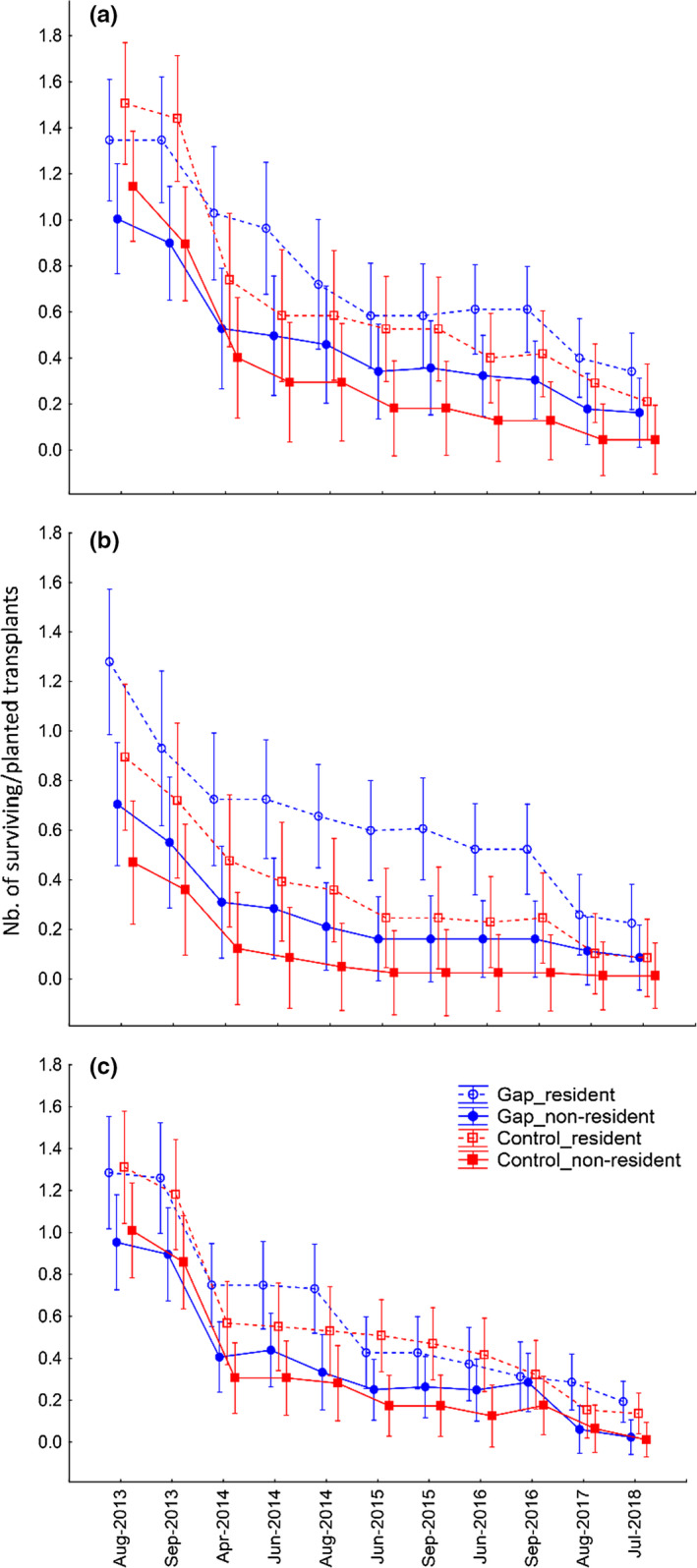
Average survival of resident and nonresident transplants in gaps and intact vegetation (control) during the experiment within each habitat: (a) *Carex acuta‐Carex panicea*, (b) *Deschmpsia caespitosa‐Carex tomentosa*, and (c) *Sesleria uliginosa‐Briza media* habitat. Error bars represent 95% confidence intervals

Both resident and nonresident sown species achieved higher rates of germination and survival in gaps compared to intact vegetation in all habitat types (Tables 1 and 2, Figure 1). Similar to sown species, transplants generally survived better in gaps than intact vegetation, but only in *Carex acuta‐Carex panicea* and *Deschampsia caespitosa‐Carex tomentosa* habitats (Table 2, Figure 2). In the *Carex acuta‐Carex panicea* habitat, gaps were initially stressful for transplants: their survival in the first year was higher in intact vegetation compared to gaps (Figure 2a). We did not observe a significant difference between transplant survival in gaps and vegetation in the *Sesleria uliginosa‐Briza media* habitat (Table 2, Figure 2c) which also displayed the lowest mean dry biomass values (Table S2). Thus, the habitat with the lowest difference between species survival in gaps and intact vegetation (Table 2) was also associated with the lowest mean biomass (Table S2).

**TABLE 1:** Repeated measures ANOVA of seedling/transplant survival of resident and nonresident species ("whole locality residence”) in gaps and control plots (Treatment) during the experiment for all habitat types (taken in one analysis together). Statistically significant (*p* < .05) results are in bold

	Seedlings			Transplants		
	*df*	*F*	*p*	*df*	*F*	*p*
Residence	**1,28**	**16.8**	**<.001**	**1,18**	**135.737**	**<.001**
Habitat	2,56	0.588	.559	**2,36**	**8.892**	**.001**
Habitat × Residence	2,56	0.988	.379	2,36	0.97	.389
Time	**11 308**	**42.914**	**<.001**	**10 180**	**136.713**	**<.001**
Time × Residence	**11 308**	**5.293**	**<.001**	10 180	0.699	.725
Treatment	**1,28**	**67.06**	**<.001**	**1,18**	**8.845**	**.008**
Treatment × Residence	**1,28**	**11.525**	**.002**	1,18	0.012	.913
Habitat × Time	**22 616**	**3.794**	**<.001**	**20 360**	**3.149**	**<.001**
Habitat × Time × Residence	22 616	0.92	.568	20 360	0.912	.572
Habitat × Treatment	2,56	2.582	.085	**2,36**	**3.303**	**.048**
Habitat × Treatment × Residence	2,56	0.533	.59	2,36	0.176	.839
Time × Treatment	**11 308**	**35.411**	**<.001**	**10 180**	**3.497**	**<.001**
Time × Treatment × Residence	**11 308**	**2.715**	**.002**	10 180	0.767	.66
Habitat × Time × Treatment	**22 616**	**2.528**	**<.001**	**20 360**	**1.783**	**.021**
Habitat × Time × Treatment × Residence	22 616	1.528	.58	20 360	0.789	.728

**TABLE 2:** Repeated measures ANOVA of seedling/transplant survival of resident and nonresident species ("whole locality residence”) in gaps and control plots (Treatment) during the experiment in different habitat types (separate analysis for each habitat type). Statistically significant results (*p* < .05) are in bold

		Residence	Time	Time × Residence	Treatment	Treatment × Residence	Time × Treatment	Time × Treatment × Residence
Seedlings								
	*df*	1,28	11 308	11 308	1,28	1,28	11 308	11 308
*Carex acuta‐Carex panicea habitat*	*F*	**17.707**	**33.83**	**4.288**	**56.99**	**10.241**	**18.064**	**2.112**
	*p*	**<.001**	**<.001**	**<.001**	**<.001**	**.003**	**<.001**	**.019**
*Deschampsia caespitosa‐Carex tomentosa habitat*	*F*	**8.901**	**26.24**	**3.249**	**51.89**	**9.041**	**31.918**	**3.633**
	*p*	**.006**	**<.001**	**<.001**	**<.001**	**.006**	**<.001**	**<.001**
*Sesleria uliginosa‐Briza media habitat*	*F*	**18.046**	**41.51**	**5.282**	**62.021**	**10.006**	**20.258**	1.245
	*p*	**<.001**	**<.001**	**<.001**	**<.001**	**.004**	**<.001**	.256
Transplants								
*Carex acuta‐Carex panicea habitat*	*df*	**1,18**	**10 180**	10 180	**1,18**	1,18	**10 180**	10 180
	*F*	**8.241**	**94.08**	0.664	**6.202**	0.059	**4.102**	0.748
	*p*	**.01**	**<.001**	.756	**.023**	.811	**<.001**	.679
*Deschampsia caespitosa‐Carex tomentosa habitat*	*df*	**1,22**	**10 220**	**10 220**	**1,22**	1,22	10 220	10 220
	*F*	**7.448**	**47.5**	**1.922**	**12.028**	0.048	1.595	0.241
	*p*	**.012**	**<.001**	**.043**	**.002**	.828	.109	.992
*Sesleria uliginosa‐Briza media habitat*	*df*	**1,22**	**10 220**	10 220	1,22	1,22	10 220	10 220
	*F*	**7.766**	**89.7**	0.759	1.803	0.227	1.594	1.819
	*p*	**.011**	**<.001**	.669	.193	.638	.11	.059

The effect of competition differed among habitats in time both for sown species and for transplants (Table 1). In cases when residency was defined across the “whole locality” (i.e., “whole locality residence”), resident species survived significantly better than nonresidents, both in gaps and in vegetation across all habitats (Tables 1 and 2). When residency was defined within a habitat (i.e., “habitat residency”), resident sown species achieved higher rates of survival than nonresidents, but this effect was only significant in the *Sesleria uliginosa‐Briza media* habitat (Table S7). Contrary, “habitat residency” influenced the survival of transplants neither in gaps nor in intact vegetation across any habitat type (Table S10).

**FIGURE 3:**
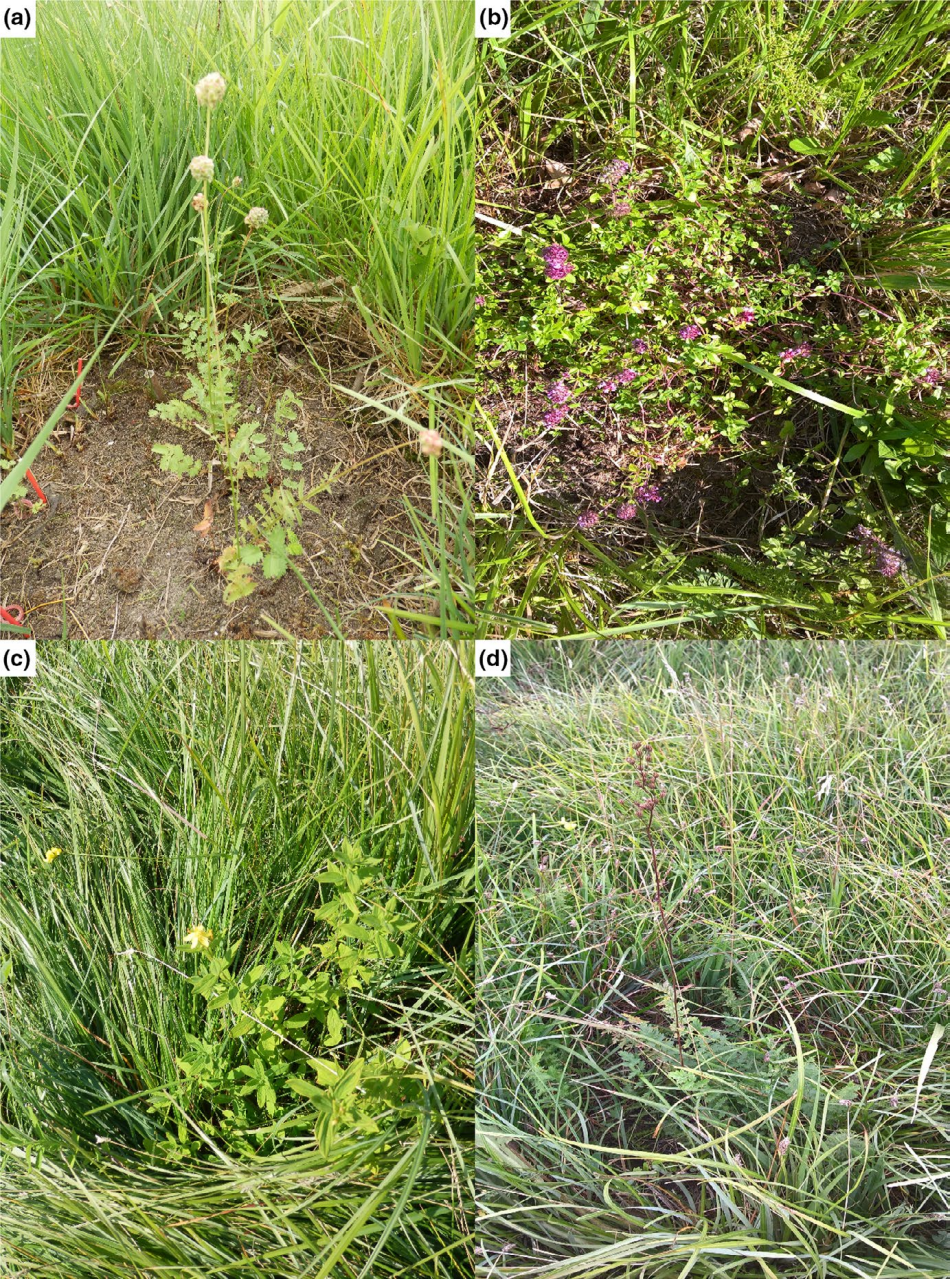
Examples of nonresident species well prospering in competition‐free gaps in 2015 ([a] *Sanguisorba minor*, [b] *Thymus pulegioides*) and in nonweeded gaps in 2018 ([c] *Hypericum hirsutum*, [d] *Filipendula vulgaris*)

If competition was removed, many nonresident species were able to establish from seeds and grow in the focal habitat (Figure 3a,b). They included species typical for much drier conditions (i.e., *Carlina aculis*, *Geranium sanguineum*, *Nardus stricta*, *Origanum vulgare*, *Sanguisorba minor*, *Thymus pulegioides*, *Trifolium montanum*) and forest species (*Hypericum hirsutum*, *Lathyrus vernus*). In the case of seed‐sowing experiment, none of these species survived within the intact vegetation. On the other hand, there were species, both resident and nonresident, which were unable to establish in intact vegetation as seeds in the seed introduction experiment, but were able to survive as transplants: *Carlina acaulis*, *Filipendula ulmaria*, *F. vulgaris*, *Geranium pratense*, *G. sanguineum*, *Hypericum hisrustum*, *Nardus stricta*, and *Sanguisorba officinalis*.

During the last two years (i.e., 2017 and 2018), when gaps were no longer controlled for weeds, the differences of survival of species in gaps and vegetation began to diminish, especially in the case of seed‐sowing experiment (Figure 1). Nevertheless, many nonresident species that became established in gaps were able to survive also in overgrown gaps. The most successful nonresident species, which survived until the summer 2018 (Figure 3c,d), were *Geranium sanguineum*, *Hypericum hirsutum*, *Nardus stricta*, *Origanum vulgare*, *Sanguisorba minor*, *Thymus pulegioides*, and *Trifolium montanum*. Several (e.g., *Hypericum hirsutum*, *Sanguisorba minor*, *Thymus pulegioides*) were even flowering in 2018. This suggests that once a species has established, its rapid competitive exclusion is difficult and unlikely.

### 3.2 Seedling/transplant survival compared with species respective Beals index values and among different habitat types

Beals index (range for our species was from 0.346 to 0.001, Table S5) was a significant predictor for seedling survival in gaps and control plots (Table S8). Seedlings of species with high Beals index (i.e., species more probable to occur in the target habitat) survived better in both gaps and intact vegetation, than species with a low Beals index (i.e., species more improbable to occur in the target habitat). Nevertheless, there were many species with low Beals index (range from 0.007 to 0.08, Table S5), and thus improbable to occur in the target habitat, which survived if competition was removed but not under competition (e.g., *Carlina aculis*, *Geranium sanguineum*, *Hypericum hirsutum*, *Lathyrus vernus*, *Nardus stricta*, *Origanum vulgare*, *Sanguisorba minor*, *Thymus pulegioides*, *Trifolium montanum*). In the case of transplants, there were also significant correlations of survival with Beals index but not so often, and what is more, there was practically no significant correlation in the *Sesleria uliginosa‐Briza media* habitat (compare Tables S8 and S11).

**FIGURE 4:**
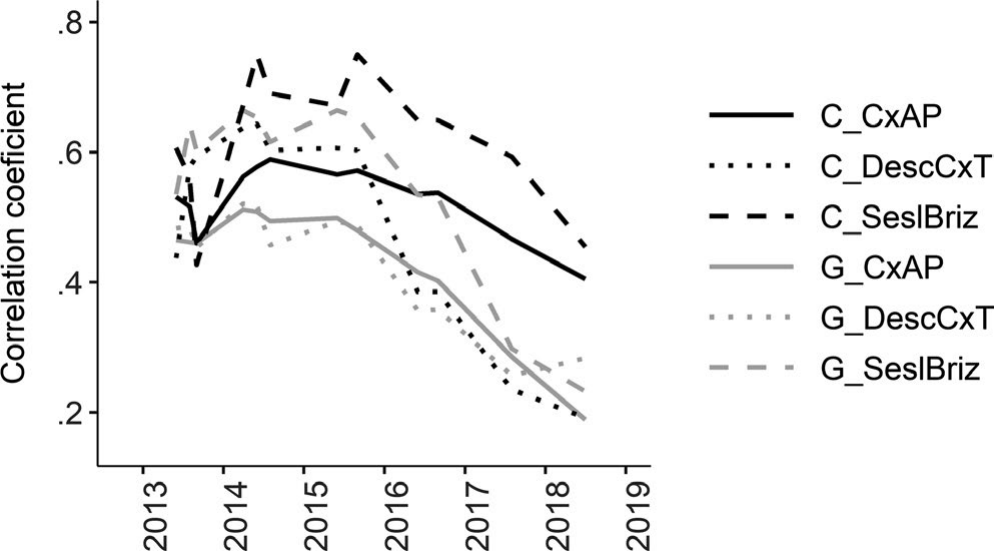
Values of Pearson's correlation coefficients between seedling survival and Beals index across years and different habitat types (*CxAP* = *Carex acuta‐Carex panicea*, *DescCxT* = *Deschampsia caespitosa‐Carex tomentosa*, *SeslBriz* = *Sesleria uliginosa‐Briza media* habitat, C = control plots—black line, G = gap—gray line)

**FIGURE 5:**
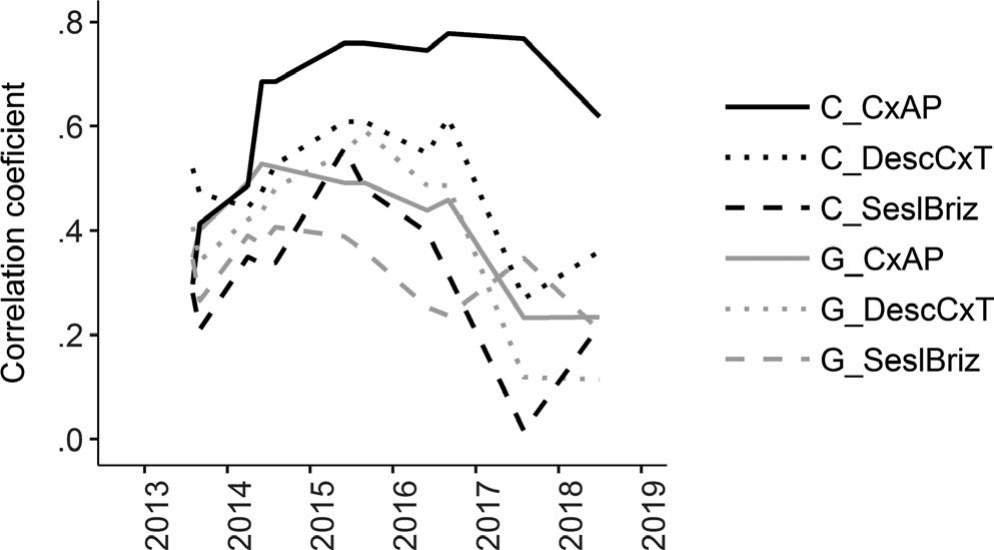
Values of Pearson's correlation coefficients between transplant survival and Beals index across years and different habitat types (*CxAP* = *Carex acuta‐Carex panicea*, *DescCxT* = *Deschampsia caespitosa‐Carex tomentosa*, *SeslBriz* = *Sesleria uliginosa‐Briza media* habitat, C = control plots—black line, G = gap—gray line)

Correlation coefficients between seedling survival and Beals index were generally higher in intact vegetation than in gaps (Figure 4). For transplants, the trend was similar but weaker, especially in the case of *Sesleria uliginosa‐Briza media* habitat where correlation coefficients were higher for intact vegetation only during 2015 and 2016. During other time points, correlations were even lower for intact vegetation than for gaps (Figure 5). Also, correlations between the ratio of survival in vegetation and in gaps and Beals index were significant and positive in the case of seedlings (Table S8). On the other hand, for transplants, they were significant only in *Carex acuta‐Carex panicea* habitat in 2015 and 2016 (Table S11). Correlations between both seedling and transplant survival in nonweeded gaps (last weeded in spring 2016) and Beals index were not significant. Similarly, correlations between the ratio of surviving both seedlings and transplants in vegetation and gaps and Beals index started to weaken once weeding stopped (Tables S8 and S11).

**FIGURE 6:**
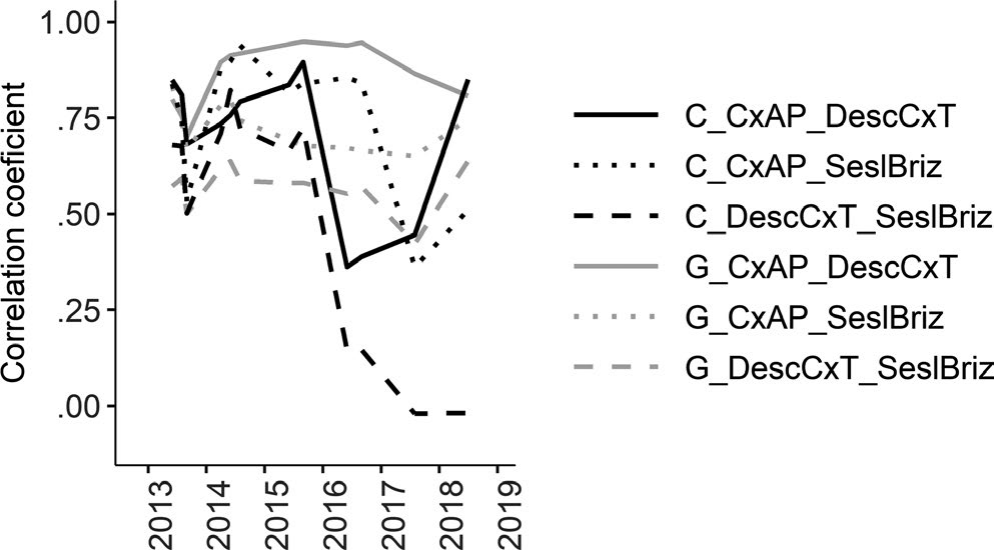
Values of Pearson's correlation coefficients between seedling survival in different habitat types (in pairs): *CxAP* = *Carex acuta‐Carex panicea*, *DescCxT* = *Deschampsia caespitosa‐Carex tomentosa*, *SeslBriz* = *Sesleria uliginosa‐Briza media* habitat, C = control plots—black line, and G = gap—gray line

Correlations of seedling survival across different habitat types (always taken in pairs) were significant, with the exception of intact vegetation between *Carex acuta‐Carex panicea* and *Sesleria uliginosa‐Briza media* habitats in 2017 and the seedling survival in vegetation between *Deschmpsia caespitosa‐Carex tomentosa* and *Sesleria uliginosa‐Briza media* habitats during the last three years (i.e., when gaps were no longer weeded) (Table S9). Correlation coefficients were higher for seedling survival in gaps when compared to control plots, especially in the case of paired *Carex acuta‐Carex panicea* and *Deschmpsia caespitosa‐Carex tomentosa* habitats (Figure 6); thus, these two habitats differed more by the biotic interactions than by abiotic environment.

## 4 DISCUSSION

### 4.1 Seed germination and survival of seedlings in contrast to transplants

Across all habitat types, sown species, both resident and nonresident, germinated and subsequently survived better in gaps than in intact vegetation. This result corresponds to many other studies where most species persisted significantly better in plots without competition (Kotorová & Lepš, 1999; Švamberková et al., 2017; Tofts & Silvertown, 2002). Zobel et al. (1998) suggested that one of the most important factors affecting species survival is the surrounding vegetation. Frei et al. (2012) highlighted the positive effect that disturbances have on the establishment of *Campanula thyrsoides* seedlings, which responded positively to cutting the surrounding vegetation and disturbing the turf. Also, in our experiment, many nonresident species with habitat preferences different from our habitats were able to establish from seeds and grow when competition was removed (similarly as in Tofts & Silvertown, 2002), but not in the intact community.

Also, transplants survived better in gaps than in intact vegetation. However, the difference between transplant survival in gaps and intact vegetation was smaller than when seeds were introduced. In the *Sesleria uliginosa‐Briza media* habitat, there were no differences between gaps and intact vegetation in the case of transplants in contrast to sown species. Aboveground biomass was there the lowest of the three habitats (Table S2), and thus, we can expect least amount of competition for light. While also this small competition was crucial for seedlings growing from seeds in the field, it was not so important problem for transplants, which are generally more resistant than seedlings (Bennett et al., 2016). There were many species that were unable to establish from seeds in intact vegetation, but survived as transplants. The biotic filter had thus a more pronounced effect on establishment from seeds, than on transplant establishment (even though they were still young individuals). In concordance with Kotorová and Lepš (1999), it seems that very early phases of seedling establishment are the most sensitive stages of many plant species and their suppression is an important filtering mechanism in the community.

Species survival was dependent on the regular weeding within gaps because both artificially created gaps and other types of naturally disturbed plots tend to become overgrown with surrounding vegetation (Puerta‐Piñero et al., 2013). Accordingly, during the last two years of our experiment (i.e., 2017 and 2018) when weeding ceased, the differences between gaps and vegetation started to decrease. Nevertheless, many nonresident species with habitat preferences different from our habitats (i.e., also species with very low Beals index and thus species very improbable to occur in target habitats) successfully established in gaps and survived also after weeding ceased and even reached their reproductive stage; confirming that competitive exclusion can be a slow process (Adler, Fajardo, Kleinhesselink, & Kraft, 2013). However, once weeding was stopped, plant mortality increased considerably, especially for seedlings. This supports the results in Gustafsson et al. (2002), which suggest that initial seedling establishment does not guarantee long‐term species survival and it is important to monitor the complete vegetation cycle of target species because sudden changes can occur in late stages of seedling establishment (Münzbergová & Herben, 2004). Also, other studies (Ehrlén, Münzbergová, Diekmann, & Eriksson, 2006; Frei et al., 2012; Houseman & Gross, 2006; Pärtel, Szava‐Kovats, & Zobel, 2013) highlight the importance of long‐term monitoring in seed addition experiments because it is possible that seeds of many species germinate and survive as seedlings for several years, but never establish a viable population (Vítová & Lepš, 2011).

### 4.2 Seedling/transplant survival compared with their respective Beals index values and among different habitat types

While the effect of species residence is a rather crude binary variable (resident/nonresident), the Beals index is based on individual species performance within an extensive set of phytosociological records from the whole region of the Czech Republic. This metric is able to distinguish between resident species regularly found within a given vegetation type and nonresident species found in similar and dissimilar habitats. In all habitat types and during the entirety of the experiment, seedling survival was positively correlated with Beals index in gaps and intact vegetation. This suggests that species are adapted to both the abiotic (correlation of survival in gaps with Beals index) and biotic conditions (correlation of survival in intact vegetation with Beals index) of particular habitats (HilleRisLambers et al., 2012). Positive correlations of species survival with Beals index was also reported by Mudrák et al. (2014), which sowed *Rhinanthus* species into a wide range of habitat types and by Milden, Münzbergová, Herben, and Ehrlén (2006) for *Succisa pratensis*. On the other hand, Münzbergová and Plačková (2010) and Frei et al. (2012) did not observe a positive relationship between Beals index and seedling survival of sown species. For transplants, the correlation of survival with Beals index was weaker than for seedlings. This again confirms that transplants are less sensitive to competition than seedlings. This supports previous observation that the primary reason for the absence of some species in a community is their inability to establish as seedlings from seeds (Tofts & Silvertown, 2002; Vítová & Lepš, 2011).

Higher correlation coefficients between Beals index and survival in intact vegetation compared to gaps and the positive correlations between the ratio of seedling survival in intact vegetation and gaps suggest that competition was the most important determinant of species community composition. These dependences were generally similar also for transplants although they were rather weak. Higher correlation coefficients of survival across habitats in gaps compared to control plots (especially in case of pair *Carex acuta‐Carex panicea* and *Deschampsia caespitosa‐Carex tomentosa* habitats) also revealed that differences in species survival within these two habitats are caused more by biotic interactions than by environmental conditions (i.e., the competition is more discriminating among species than the effect of the abiotic environment). Bar‐Massada (2015) suggested that biotic interactions are the most important drivers of species co‐occurrence, although their effect could be influenced by environmental heterogeneity. Many other studies highlight the importance of biotic interactions in determining species community composition and the necessity to incorporate them into models (Boulangeat, Gravel, & Thuiller, 2012; Morales‐Castilla, Matias, Gravel, & Araújo, 2015; Myers & Harms, 2011; Pollock et al., 2014; Wisz et al., 2013). Conversely, D’Amen et al. (2018) suggested that environmental filtering and dispersal limitation are more important drivers of species co‐occurrence than biotic interactions, but this conclusion was based on the analyses of observational data and the use of null models. In our view, without direct experimental manipulation of biotic interactions, it is difficult to distinguish the direct effect of environment from environmentally modified biotic interactions (Cadotte & Tucker, 2017).

## 5 CONCLUSIONS

Many nonresident species very improbable to occur in the target habitats (i.e., with low Beals index) were able to perform well in competition‐free gaps, but were unable to survive in intact vegetation. These species were thus not limited by the abiotic conditions, but by competition with neighboring plants. Although the appropriate abiotic conditions are important for seedling survival, our experiment suggests that biotic interactions are likely the most important determinants of plant species community composition and operate mainly through prevention of establishment of the “unsuitable” species. Although Beals index is a good predictor of species survival in plant communities, we should be careful to use it as species pool determinant, especially in disentangling the effect of abiotic and biotic filter on species community composition. If we define the community species pool as a set of species able to survive and reproduce in given abiotic environment (Butaye et al., 2001), the set of species will be much wider than predicted by Beals index (and generally any comparative method) because we extend the species pool about species otherwise excluded by biotic filter. Comparative methods generally exclude species which are not able to withstand the competition from species pool. If we compare the actual community composition with this species pool with the aim to disentangle the importance of biotic and abiotic factors, we would underestimate the effect of competition because species affected by competition are already excluded from this species pool.

## CONFLICT OF INTEREST

The authors declare no competing interests.

## AUTHOR CONTRIBUTIONS

**Eva Švamberková:** Conceptualization (supporting); Formal analysis (equal); Investigation (lead); Methodology (supporting); Writing‐original draft (lead); Writing‐review & editing (equal). **Jan Lepš:** Conceptualization (lead); Formal analysis (equal); Investigation (supporting); Methodology (lead); Supervision (lead); Writing‐review & editing (equal).

## Supporting information

Supplementary material

## Data Availability

Data associated with this study are available from the Dryad Repository (https://doi.org/10.5061/dryad.fqz612jq9).
