# Supplementary material for: Experimental assessment of biotic and abiotic filters driving community composition
Source: Ecol Evol. 2020 Jun 13;10(14):7364–76. doi: 10.1002/ece3.6461 (PMC7391324; doi:10.1002/ece3.6461)
Supplement: Supplementary file 1 — Supplementary material [file ECE3-10-7364-s001.pdf]

Appendices

Appendix 1 – Study site characteristics

Meteorological data

Table S1. Mean annual temperature and total annual precipitation in Strašovský rybník from 2013 to 2018 (based on the measurements of the Czech Hydrometeorological Institute in a meteorological station in Mokošín, 12 km from Strašovský rybník).

	2013	2014	2015	2016	2017	2018
Mean annual temperature [°C]	9.5	11.1	11.1	10.3	10.1	11.5
Total annual precipitation [mm]	559.8	548	451.1	394.1	594.5	375.1

Measurement of moisture

From July 2013 to October 2016, we measured the volumetric soil moisture both in gaps and in the intact vegetation using TOMST datallogers TMS3 in 15 minutes period. Then we calibrated data using TMS3Calibr (TOMST 2013) and counted mean daily soil volumetric moisture (Fig. S1).

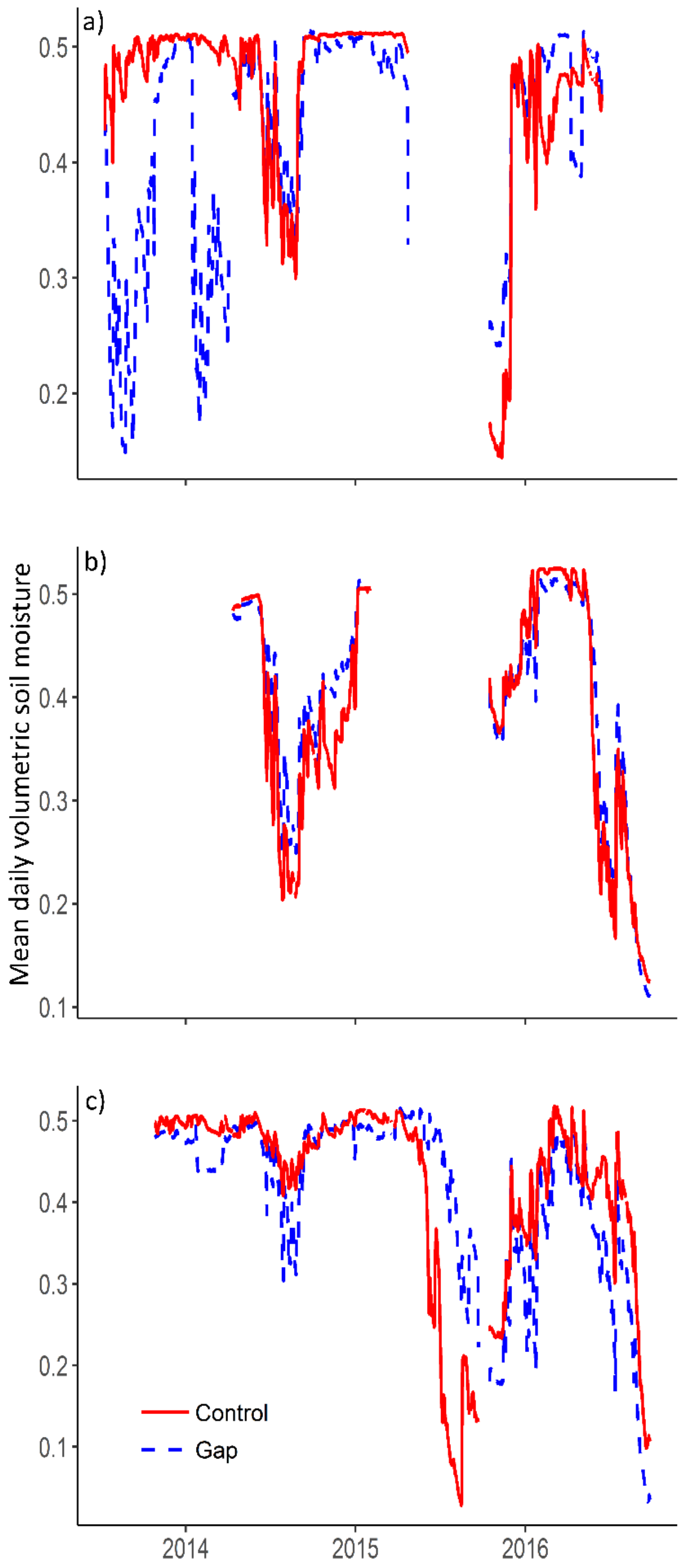

a)
b)
c)
Mean daily volumetric soil moisture
0.5
0.4
0.3
0.2
0.1
2014
2015
2016
Control
Gap

Fig. S1. Mean daily volumetric soil moisture in gaps and control plots in a) *Carex acuta-Carex panicea*, b) *Deschampsia cespitosa-Carex tomentosa*, c) *Sesleria uliginosa-Briza media* habitat.

Species composition data

In June 2014, five phytosociological relevés (3x3m) on each habitat type were conducted. We recorded the cover of present species in each relevé. Species nomenclature refers to Kubát et al. (2002).

To show different species composition of each study habitat type, we conducted a Redundancy analysis (RDA) in CANOCO 5 (ter Braak and Šmilauer 2012) with centring and no standardisation neither by specie nor by samples (Fig. S2). Species composition (cover estimates) was used as response variables and different habitat types (*Carex acuta-Carex panicea*, *Deschampsia cespitosa-Carex tomntosa* and *Sesleria uliginosa-Briza media* habitat) as explanatory variables.

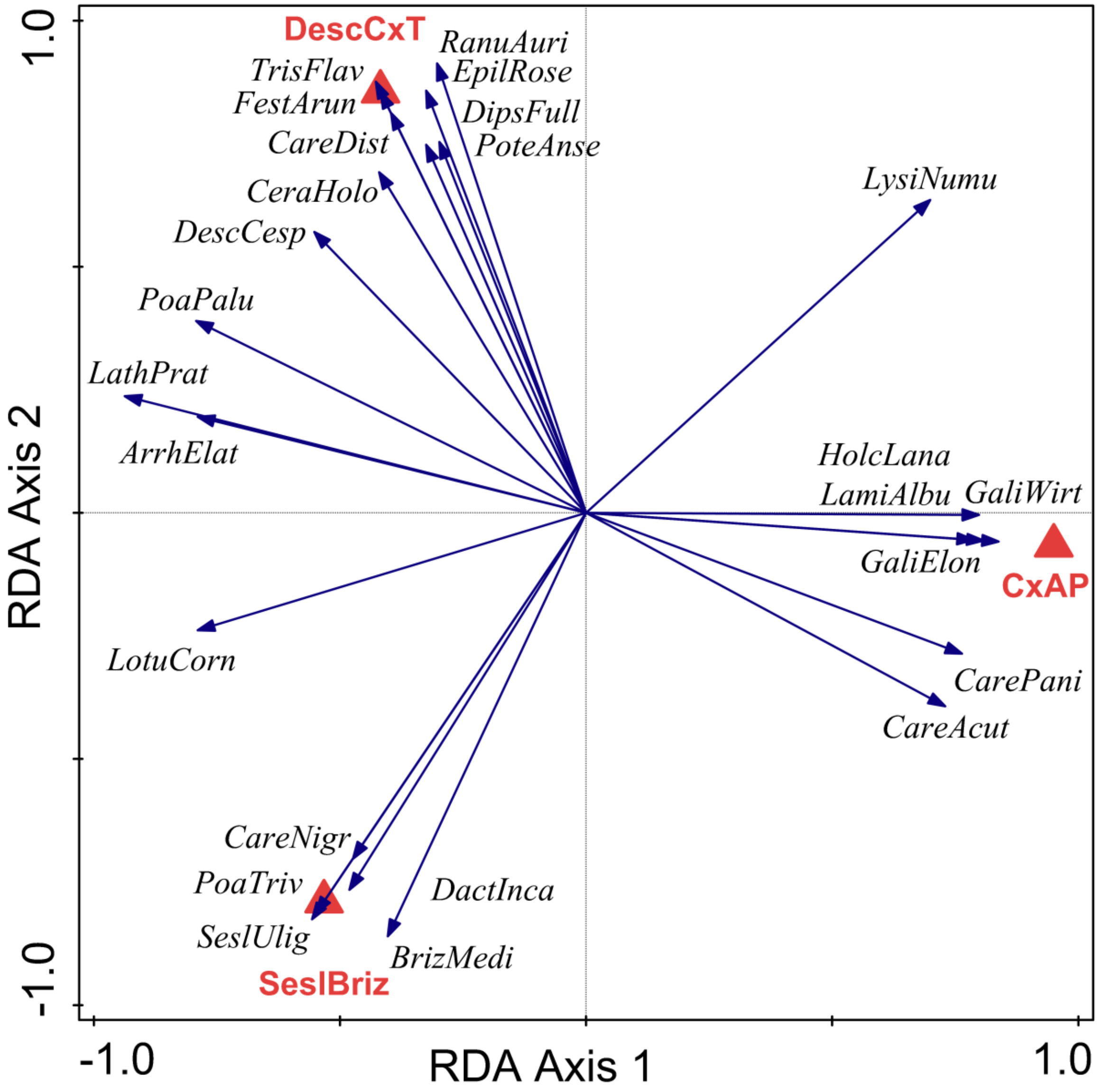

Fig. S2. RDA of species composition on different habitat types (*CxAP* = *Carex acuta-Carex panicea*, *DescCxT* = *Deschampsia cespitosa-Carex tomentosa*, *SeslBriz* = *Sesleria uliginosa-Briza media* habitat). Adjusted explained variation = 50.72%, pseudo-F = 8.2, p = 0.002. Red triangles mark different habitat types and blue arrows individual species. *ArrhElat – Arrhenatherum elatius, BrizMedi – Briza media, CareAcut – Carex acuta, CareDist – Carex disticha, CareNigr – Carex nigra, CarePani – Carex panicea, CeraHolo – Cerastium*

holosteioides, DactInca – Dactylorhiza incarnata, DescCesp – Deschampsia cespitosa, DipsFull – Dipsacus fullosa, EpilRose – Epilobium roseum, FestArun – Festuca arundinacea, GaliElon – Galium elongatum, GaliWirt - Galium wirtgenii, HolcLana – Holcus lanatus, LamiAlbu – Lamium album, LathPrat – Lathyrus pratensis, LotuCorn – Lotus corniculatus, LysiNumu – Lysimachia nummularia, PoaPalu – Poa palustris, PoaTriv – Poa trivialis, PoteAnse – Potentilla anserina, RanuAuri – Ranunculus auricomus, SeslUlig – Sesleria uliginosa, TrisFlav – Trisetum flavescens.

<u>Biomass samples</u>

In June 2019, we cut the biomass from five 0.5 x 0.5 m plots in each experimental habitat type. The biomass from each plot was dried at 110°C until its mass was considered to become constant. Then we weighed the dry biomass of each sample. Subsequently, we calculated the mean biomass weight from five samples for each habitat type.

Differences in biomass among the three habitat types were tested using one-way ANOVA with post-hoc comparisons using Tukey HSD method in Statistica 13 (StatSoft, 2015). Homogeneity of variances was tested using Bartlett test.

There were significant differences in biomass among different habitat types ($F_{2,12}$ = 11.458, p = 0.002) and post-hoc tests have demonstrated significant difference between biomass on *Carex acuta-Carex panicea* and *Sesleria uliginosa-Briza media* habitat type (p = 0.023) and on *Deschampsia cespitosa-Carex tomntosa* and *Sesleria uliginosa-Briza media* habitat type (p = 0.002). The lower mean dry mass of biomass was recorded on *Sesleria uliginosa-Briza media*

habitat type (Table S2). There was no significant difference between the biomass on *Carex acuta-Carex panicea* and *Deschampsia cespitosa-Carex tomntosa* habitat ($p = 0.284$, Table S2).

Table S2. Mean dry weight of biomass from different habitat types and their standard deviation. Letters *a* and *b* illustrate the differences in biomass among different habitat types in post-hoc test of one-way ANOVA.

Habitat type	Mean dry mass of biomass [g/0.25m2]	Standard deviation [g/0.25m2]
Carex acuta-Carex panicea	90.14 *a*	6.06
Deschampsia cespitosa-Carex tomntosa	98.87 *a*	11.35
Sesleria uliginosa-Briza media	73.158 *b*	3.68

Soil samples

In August 2014, we took five soil samples (5cm of diameter, 20 cm deep) from six plots (0.5x0.5m) from each habitat type. Five samples from each plot were mixed together and subsequently laboratory analysed.

Differences in soil characteristics among the three habitat types were tested by the same analysis as in case of biomass. P-PO_4 and granularity 50-100% values were log-transformed to achieve, resp. to improve homogeneity of variances required by the F-test in one-way ANOVA.

There were significant differences in all soil characteristics among different habitat types with exception of the soil granularity 10-50 and 50-100 μm (Table S3). Post-hoc tests have demonstrated some significant differences between some soil characteristics on different habitat types (Table S3).

Table S3. Soil sample characteristics (mean values from 6 plots for each habitat type) in different habitat types and results of their ANOVA. Letters *a*, *b* and *c* illustrate the differences in soil characteristics among different habitat types in post-hoc test of one-way ANOVA.

	Carex acuta-*Carex panicea* habitat	*Deschampsia cespitosa*-*Carex tomntosa* habitat	*Sesleria uliginosa*-*Briza media* habitat	$F_{2,15}$	p
pH H_2O	7.61 *a*	7.76 *a*	7.99 *b*	15.89	**< 0.001**
pH KCl	7.17 *a*	7.32 *b*	7.55 *c*	28.3	**< 0.001**
conductivity [μS/cm]	758.00 *b*	224.50 *a*	299.50 *a*	147.63	**< 0.001**
organic contain (loss by annealing) [%]	25.31 *b*	18.79 *a*	18.91 *a*	17.02	**< 0.001**
dry matter [%]	93.41 *b*	96.04 *a*	96.30 *a*	46.5	**< 0.001**
N-NH_4 [mg/kg]	10.355 *b*	2.583 *a*	17.010 *c*	56.08	**< 0.001**
P-PO_4 [mg/kg]	10.799 *a*	19.815 *b*	8.573 *a*	10.27	**0.002**
P [mg/kg]	545.943 *a*	572.311 *a*	363.287 *b*	17.8	**< 0.001**
N [%]	1.20 *a*	1.03 *b*	1.22 *a*	17.81	**< 0.001**
C [%]	12.58 *b*	11.37 *a*	14.73 *c*	53.81	**< 0.001**
K [mg/kg]	245.700 *b*	313.083 *c*	107.290 *a*	56.68	**< 0.001**
granularity [μm]					
0-2 [%]	16.51 *b*	13.90 *ab*	10.75 *a*	8.38	**0.004**
2-10 [%]	35.03 *a*	32.81 *a*	26.50 *b*	6.84	**0.008**

10-50 [%]	21.31 *a*	14.35 *a*	17.82 *a*	1.77	0.205
50-100 [%]	0.32 *a*	2.76 *a*	2.93 *a*	2.4	0.125
100-2000 [%]	26.83 *b*	36.68 *a*	42.14 *a*	12	**0.001**

Ellenberg indicator values

Community weighted mean (CWM) for each habitat type was calculated according to Garnier et al. (2004) using species cover from five phytosociological relevés for each habitat type from June 2014 weighted by Ellenberg indicator values for light, moisture and nutrients. Differences in CWM among the three habitat types were tested by the same analysis as in case of biomass and soil characteristics. CWM of Ellenberg indicator values for moisture was log-transformed to improve homogeneity of variances required by the F-test in one-way ANOVA.

There were significant differences in CWM in case of all Ellenberg indicator values among different habitat types (Table S4). Post-hoc tests have demonstrated some significant differences in CWM of some Ellenberg indicator values on different habitat types (Table S4).

Table S4. Community weighted mean (CWM) of Ellenberg indicator values (for light, moisture and nutrients) for different habitat type (mean from five phytocenological rélevés) and results of their ANOVA. Letters *a* and *b* illustrate the differences in CWM among different habitat types in post-hoc test of one-way ANOVA.

	Carex acuta-Carex panicea habitat	*Deschampsia cespitosa-Carex tomntosa* habitat	*Sesleria uliginosa-Briza media* habitat	$F_{2,12}$	p
CWM_Light	6.68 *a*	6.71 *a*	7.27 *b*	25.68	**< 0.001**
CWM_Moisture	6.92 *b*	6.31 *a*	6.67 *ab*	9.22	**0.004**
CWM_Nutrients	4.47 *a*	4.72 *a*	3.37 *b*	76.38	**< 0.001**

References:

Garnier E., Cortez J., Billes G., Navas M. L., Roumet C., Debussche M., Laurent G., Blanchard A., Aubry D., Bellmann A., Neill C., & Toussaint J. P. (2004): Plant functional markers capture ecosystem properties during secondary succession. – Ecology. 85: 2630-2637.

Kubát K., Hrouda L., Chrtek J. jun., Kaplan Z., Kirschner J. & Štěpánek J. (eds.) (2002): Klíč ke květené České republiky [Nomenclature key to determination of the Czech Republic flora]. – Academia, Praha.

ter Braak C. J. F. & Šmilauer P. (2012): Canoco reference manual and user's guide: Software for ordination (Version 5.0). Microcomputer Power, Ithaca.

StatSoft (2015): STATISTICA (data analysis software system), version 13. – StatSoft Inc. URL: [www.statsoft. com].

TOMST (2013): TMS3Calibr TOMST Measuring System - Station for spatially distributed measurements of soil moisture and ambient temperature in vast fields. – TOMST s.r.o., Prague. URL: [http://www.tomst.com/tms].

Apendix 2 – Additional tables and figures

Table S5. Beals index and residence of species sown in seed introduction experiment.

	Beals index			Residence			
Species	*Carex acuta-Carex panicea* habitat	*Deschampsia cespitosa-Carex tomntosa* habitat	*Sesleria uliginosa-Briza media* habitat	*Carex acuta-Carex panicea* habitat	*Deschampsia cespitosa-Carex tomntosa* habitat	*Sesleria uliginosa-Briza media* habitat	Whole locality
Anthoxanthum odoratum	0.326	0.313	0.346	resident	non-resident	resident	resident
Aphanes arvensis	0.001	0.003	0.001	non-resident	non-resident	non-resident	non-resident
Arabis glabra	0.002	0.002	0.002	non-resident	non-resident	non-resident	non-resident
Bistorta major	0.116	0.121	0.105	non-resident	non-resident	non-resident	non-resident
Bupleurum falcatum	0.029	0.030	0.031	non-resident	non-resident	non-resident	non-resident
Campanula persicifolia	0.016	0.019	0.020	non-resident	non-resident	non-resident	non-resident
Carlina acaulis	0.050	0.059	0.070	non-resident	non-resident	non-resident	non-resident

Cirsium acaule	0.015	0.016	0.032	non-resident	non-resident	non-resident	non-resident
Filipendula ulmaria	0.221	0.190	0.213	non-resident	non-resident	non-resident	resident
Filipendula vulgaris	0.040	0.042	0.063	non-resident	non-resident	non-resident	resident
Galium boreale	0.080	0.086	0.131	resident	resident	resident	resident
Geranium pratense	0.069	0.085	0.063	non-resident	non-resident	non-resident	resident
Geranium sanguineum	0.007	0.007	0.007	non-resident	non-resident	non-resident	non-resident
Hypericum hirsutum	0.003	0.003	0.002	non-resident	non-resident	non-resident	non-resident
Lathyrus vernus	0.012	0.014	0.011	non-resident	non-resident	non-resident	non-resident
Lotus corniculatus	0.165	0.192	0.254	resident	resident	resident	resident
Lycopus europeus	0.065	0.043	0.037	non-resident	non-resident	non-resident	resident
Lychnis flos-cuculi	0.308	0.281	0.280	resident	resident	resident	resident
Malva neglecta	0.001	0.002	0.001	non-resident	non-resident	non-resident	non-resident
Melica nutans	0.017	0.020	0.018	non-resident	non-resident	non-resident	non-resident
Nardus stricta	0.098	0.086	0.105	non-resident	non-resident	non-resident	non-resident
Origanum vulgare	0.016	0.018	0.018	non-resident	non-resident	non-resident	non-resident
Plantago lanceolata	0.276	0.326	0.325	non-	non-resident	resident	resident

				resident			
Prunella vulgaris	0.176	0.171	0.215	non-resident	non-resident	resident	resident
Sanguisorba minor	0.059	0.067	0.074	non-resident	non-resident	non-resident	non-resident
Sanguisorba officinalis	0.301	0.292	0.337	resident	resident	resident	resident
Scutellaria galericulata	0.040	0.024	0.027	non-resident	non-resident	non-resident	resident
Thymus pulegioides	0.069	0.083	0.103	non-resident	non-resident	non-resident	non-resident
Trifolium montanum	0.030	0.033	0.047	non-resident	non-resident	non-resident	non-resident
Viola hirta	0.049	0.051	0.072	non-resident	non-resident	non-resident	non-resident

Table S6. Transplants planted in different habitat types (in two replications) and their initial characteristics in time of planting. Transplant identity characterises the numeric mark of planted transplant (1, 2, 3) and treatment (g = gap, c = control plot) where it was planted. Transplants excluded from the experiment because their pre-growth was unsuccessful are marked as “-“.

		Carex acuta-Carex panicea 1		*Carex acuta-Carex panicea 2*		*Deschampsia cespitosa-Carex tomntosa 1*		*Deschampsia cespitosa-Carex tomntosa 2*		*Sesleria uliginosa-Briza media 1*		*Sesleria uliginosa-Briza media 2*	
	Transplant identity	Height [cm]	Nb. of leaves	Height [cm]	Nb. of leaves	Height [cm]	Nb. of leaves	Height [cm]	Nb. of leaves	Height [cm]	Nb. of leaves	Height [cm]	Nb. of leaves
Anthoxanthum odoratum	1g	4.6	1	7.7	2	3.7	3	4.7	2	4	3	3.5	3
	2g	3.5	2	4.4	3	6.5	2	8.9	2	3.1	3	8.8	5
	3g	2.1	2	3.6	2	8	4	9.1	3	4.3	3	4.7	2
	1c	3.2	2	9.2	4	5.1	3	10.7	2	3.2	2	3	3
	2c	3.7	4	5	2	4.8	5	5.1	2	2.6	2	9.1	5
	3c	3.7	2	5.6	5	6.5	3	5	2	4.8	3	6.6	3
Aphanes arvensis	1g	1	4	2	4	1	4	1.1	7	1.7	7	1.2	5
	2g	1	3	2.2	5	1.3	5	0.5	4	1.5	7	1	4
	3g	1.1	4	1.1	6	1.5	6	1.3	5	1.5	8	1.3	5
	1c	1	3	0.9	3	1.2	7	0.6	3	1.4	7	1	4
	2c	0.8	4	1.1	5	1.5	7	1.2	6	1.6	6	1.5	6
	3c	1.1	6	0.7	4	1	6	1.1	6	1.6	6	1.6	4
Arabis glabra	1g	1	4	0.6	4	0.5	4	0.2	3	0.8	4	1	3
	2g	0.7	6	0.7	6	1	5	0.4	4	0.6	2	0.5	3
	3g	0.7	4	0.7	4	0.7	5	0.7	4	0.9	3	0.8	4
	1c	0.7	3	0.9	4	0.6	4	0.5	3	0.7	3	0.6	3
	2c	0.8	4	0.7	6	0.3	6	0.4	4	0.6	2	0.5	5
	3c	0.7	4	1.2	6	0.5	5	0.3	6	0.7	3	0.5	4
Bistorta major	-	-	-	-	-	-	-	-	-	-	-	-	-
Bupleurum falcatum	-	-	-	-	-	-	-	-	-	-	-	-	-
Campanula persicifolia	1g	1.1	2	0.2	2	0.7	2	0.5	2	0.8	1	0.6	2
	2g	1.2	2	0.6	2	0.5	2	0.2	2	0.5	2	0.4	2
	3g	1.3	2	0.5	2	0.4	3	0.6	2	0.6	2	0.5	3
	1c	1.5	3	0.9	2	0.5	2	0.3	2	0.7	2	0.5	2
	2c	1.7	3	0.2	1	0.5	2	0.3	4	0.3	2	0.6	2
	3c	1	2	0.3	2	0.3	2	0.2	2	0.8	2	0.5	4
Carlina acaulis	1g	1.9	4	3.8	4	1.8	4	2.1	4	3	4	3	3
	2g	3.4	4	2.5	4	2.5	4	1.9	4	3.5	4	1.7	3

	3g	2.9	4	2.7	4	2	3	3.3	4	3.5	3	1.5	4
	1c	2.6	4	3.6	4	3.5	4	2.3	4	2.5	3	3.1	4
	2c	2.1	4	2.6	4	3.5	4	1.7	3	3.2	4	3.2	4
	3c	2	4	3.4	4	2.5	4	2.4	4	2.7	3	2	4
Cirsium acaule	1g	-	-	-	-	1.5	2	-	-	1.8	1	0.5	0
	2g	-	-	-	-	2.3	2	-	-	2	2	1.9	2
	3g	-	-	-	-	-	-	-	-	1.1	1	1.4	2
	1c	-	-	-	-	2	2	-	-	1.5	2	2.1	4
	2c	-	-	-	-	1.7	2	-	-	2.4	2	3.1	3
	3c	-	-	-	-	-	-	-	-	2.5	4	2.3	2
Filipendula ulmaria	1g	-	-	-	-	1	2	-	-	0.7	2	0.9	2
	2g	-	-	-	-	0.8	2	-	-	1	2	0.7	2
	3g	-	-	-	-	0.7	2	-	-	0.9	2	1	2
	1c	-	-	-	-	0.9	2	-	-	1.2	3	1	2
	2c	-	-	-	-	0.6	3	-	-	0.6	2	0.8	2
	3c	-	-	-	-	1.1	3	-	-	0.6	1	0.8	2
Filipendula vulgaris	1g	1	4	0.9	3	1	4	1	3	0.7	3	1.1	4
	2g	1.1	3	0.9	2	1.3	4	1.6	5	0.7	3	0.8	3
	3g	1	3	0.6	3	0.7	3	1	3	1	3	1	3
	1c	0.8	3	1	4	1.2	4	0.8	3	1	3	1.2	2
	2c	1.3	4	1	3	1.8	4	0.9	3	0.8	2	1.5	3
	3c	1.2	3	0.7	3	2	4	1.3	3	0.4	4	1	3
Galium boreale	1g	1.2	8	0.7	4	1.5	12	1.5	4	1.5	6	2	19
	2g	2.2	8	2.1	5	1.7	9	1.5	8	1	10	2.5	11
	3g	1.5	6	1.4	7	2.3	12	1.2	6	1.5	13	2.3	6
	1c	0.9	6	2.5	8	1.7	7	1.8	9	1.9	14	1.7	8
	2c	1.3	8	1.1	8	2.5	10	1	6	1.5	8	2.5	13
	3c	1.9	8	0.8	4	2.5	7	2.3	8	0.5	4	1	5
Geranium pratense	1g	8.1	1	8	1	8	1	5.7	3	9	1	11	1
	2g	8.7	1	7.6	0	4.5	1	7.6	2	8.5	1	11.5	1
	3g	11.1	2	5.1	1	5.5	1	7.4	1	6.8	1	10.5	1
	1c	6	2	8.5	1	6	1	14.6	1	8	1	12.5	1
	2c	7.3	1	6	1	8.5	1	5.5	1	6	1	10.5	2
	3c	6.8	1	5.6	1	8	1	3.4	1	7.5	1	8	1
Geranium sanguineum	1g	5	2	6.3	1	7	2	3.3	2	4	2	2.5	1
	2g	4.3	2	6.1	2	6	1	5.8	1	6	1	3.5	1
	3g	5.5	2	8	1	5	2	4	2	7	1	4.5	1
	1c	6.5	1	7.2	2	6.7	2	4.2	1	5.5	2	5.5	2
	2c	4.9	1	6.3	1	5	2	4.1	1	5.7	2	6	2
	3c	3.7	1	4.8	1	3.5	2	3.5	1	4.5	2	6.5	1
Hypericum hirsutum	1g	0.6	4	0.6	4	1	8	0.2	4	0.1	4	0.2	4
	2g	0.7	4	0.2	2	0.2	2	0.3	4	0.2	6	0.5	4
	3g	0.6	4	0.6	4	1	6	0.4	4	0.1	4	0.5	4
	1c	0.6	2	0.7	4	0.7	6	0.5	6	0.1	2	1	6
	2c	0.6	4	0.6	4	0.5	6	0.4	6	0.1	4	1	6

	3c	0.6	4	0.7	2	1	4	0.2	4	0.1	4	0.5	4
Lathyrus vernus	1g	4.2	2	1.2	1	2.2	2	3.5	2	2	1	5.5	2
	2g	1.9	1	2	1	2.8	2	2.8	2	3	1	3.5	4
	3g	3.1	2	2.6	1	2	1	2.7	2	4.1	1	5.5	2
	1c	6	2	2	2	2.1	1	5.6	2	2	2	3.5	2
	2c	3	2	2.9	1	8	3	3.8	2	3.7	2	3.5	2
	3c	3.7	2	3.4	1	3	3	5.5	2	2.2	1	6	2
Lotus corniculatus	1g	14	18	2.9	2	3.5	7	7.5	10	2.8	2	7.5	6
	2g	1.9	11	2.6	4	6	7	3.2	5	1.5	2	8.5	5
	3g	3.1	5	2.3	4	4.5	5	4.7	5	1.8	3	8	5
	1c	6	3	2.5	4	8	12	3.2	4	2.1	4	7.5	7
	2c	3	7	2.1	3	6	8	6.7	11	3.5	3	14	12
	3c	3.7	3	3.4	7	10.2	8	2.5	7	2.7	4	12.5	14
Lycopus europeus	-	-	-	-	-	-	-	-	-	-	-	-	-
Lychnis flos-cuculi	1g	0.8	6	0.6	4	1	4	0.5	4	0.5	4	1	6
	2g	0.7	6	0.6	4	1	6	0.6	4	0.5	4	1	6
	3g	0.7	4	0.7	4	1	7	0.6	6	0.4	5	0.7	4
	1c	0.8	4	0.8	4	1	6	0.5	4	0.3	6	1	4
	2c	0.6	6	0.6	4	1.3	8	0.3	4	0.2	4	1	6
	3c	0.6	4	0.8	6	1	5	0.6	6	0.5	4	0.5	4
Malva neglecta	1g	-	-	-	-	1.5	1	-	-	1.4	1	0.5	1
	2g	-	-	-	-	1	1	-	-	0.9	0	0.5	1
	3g	-	-	-	-	-	-	-	-	1	1	0.6	2
	1c	-	-	-	-	1	2	-	-	1.5	1	0.5	1
	2c	-	-	-	-	0.7	1	-	-	0.8	2	1.3	1
	3c	-	-	-	-	-	-	-	-	1	0	1	2
Melica nutans	-	-	-	-	-	-	-	-	-	-	-	-	-
Nardus stricta	1g	3	4	3.3	4	2.3	5	5	2	2.8	4	2.2	3
	2g	2.6	2	3.1	3	4.5	4	3.1	3	3.6	5	4.2	2
	3g	3.5	4	3.1	3	3.5	4	9	3	3.2	3	3.3	2
	1c	3.2	3	2.7	3	3	5	2.9	3	2	4	3.2	4
	2c	3.3	4	4.7	3	3	3	10.7	3	2.6	4	2	2
	3c	2.4	4	3	3	2.8	4	20.3	3	3	4	2.6	4
Origanum vulgare	1g	0.6	4	0.7	4	1.5	4	0.3	4	0.3	2	0.5	6
	2g	0.8	4	0.8	4	1	3	0.4	6	0.2	4	0.7	4
	3g	0.9	4	0.6	4	0.8	4	0.2	4	0.5	4	0.4	4
	1c	0.6	4	0.8	6	0.3	4	0.4	4	0.3	6	0.4	4
	2c	0.6	4	0.6	2	0.6	4	0.7	6	0.3	4	0.5	4
	3c	0.6	2	0.6	2	1	4	0.6	4	0.5	5	0.8	4
Plantago lanceolata	1g	4.4	2	3.4	2	5.9	1	4	3	2.5	2	8.2	4
	2g	5.2	1	4.3	1	5	2	4.1	1	5.6	1	8.5	3
	3g	5.4	2	3.1	1	8.5	2	5.9	2	3.5	2	11	4
	1c	7.6	2	4.4	1	4.5	1	2.6	2	5.7	2	7.5	4
	2c	4.4	1	6.3	2	6.5	1	5.9	2	3.7	1	7.8	4

	3c	5	2	3.9	1	8.5	2	4	1	5	2	2.5	3
Prunella vulgaris	1g	0.8	4	1.2	4	1.5	6	0.2	4	1	4	0.5	6
	2g	1.1	4	1.3	4	1	4	0.3	4	0.7	4	1	6
	3g	0.7	4	1.7	6	0.5	4	0.9	4	0.3	4	0.9	8
	1c	0.6	4	1.3	4	0.5	4	0.4	4	0.6	4	1	4
	2c	0.7	4	1.2	4	0.7	6	0.5	4	0.5	2	1.3	6
	3c	1.2	4	1.3	4	0.8	6	0.8	4	0.9	4	1	4
Sanguisorba minor	1g	11.2	2	7.7	3	6.5	3	4.7	5	8.5	3	7	3
	2g	10.5	3	8.4	4	7.8	4	7.9	4	6.1	2	9	2
	3g	12.4	2	7.7	4	6.5	3	8.8	4	6.3	3	4.9	2
	1c	9.4	3	11.1	4	8.5	5	8.8	3	12.5	2	4.8	5
	2c	9.3	2	6.2	3	9.5	3	5.6	3	8.5	4	8.7	2
	3c	6	3	6.6	3	5.5	2	5.4	3	7	2	9.3	3
Sanguisorba officinalis	1g	6.6	2	5.4	2	4.8	2	4.1	2	5	2	3.5	2
	2g	3.4	1	4.8	1	3	1	3.2	2	8	2	6	1
	3g	4.6	2	4.9	1	2.5	2	4.2	2	7	2	3.7	1
	1c	6.9	2	5.2	2	4.5	2	3.8	1	5.5	3	6.1	3
	2c	7.4	2	2.9	2	3.7	2	2.5	2	6	2	4	2
	3c	4.6	2	4.4	1	3.5	2	5.1	2	6.5	2	5.5	2
Scutellaria galericulata	-	-	-	-	-	-	-	-	-	-	-	-	-
Thymus pulegioides	1g	-	-	-	-	1.5	6	-	-	0.5	4	0.7	6
	2g	-	-	-	-	1.8	4	-	-	0.3	2	1.5	3
	3g	-	-	-	-	0.3	0	-	-	1.3	4	0.4	2
	1c	-	-	-	-	1.3	6	-	-	2.5	6	2	10
	2c	-	-	-	-	0.5	4	-	-	0.9	6	1.1	6
	3c	-	-	-	-	0.7	8	-	-	1	10	1.8	10
Trifolium montanum	1g	0.6	2	0.2	3	0.8	1	0.6	2	0.5	3	1.1	3
	2g	0.7	3	0.7	2	1	3	0.3	4	0.8	1	1	2
	3g	0.7	1	0.3	2	1.8	2	0.6	3	1.2	3	1.6	3
	1c	1	3	0.5	3	1.3	1	0.3	2	1	2	0.6	2
	2c	0.9	2	0.6	3	1.8	6	0.5	3	1	3	1.1	2
	3c	0.8	2	0.2	1	1	2	1.5	2	0.8	2	0.9	2
Viola hirta	-	-	-	-	-	-	-	-	-	-	-	-	-

Table S7: Repeated Measurement ANOVA of seedling survival of resident and non-resident species (“habitat residence”) in gaps and control plots (Treatment) during the experiment in different habitat types (separate analysis for each habitat type). Statistically significant results are in bold.

		Carex acuta-Carex panicea habitat		*Deschampsia caespitosa-Carex tomentosa habitat*		*Sesleria uliginosa-Briza media habitat*	
	Degree of freedom	F	p	F	p	F	p
Residence	1,28	2.927	0.098	1.834	0.186	**23.968**	**<0.001**
Time	11,308	**28.358**	**<0.001**	**21.891**	**<0.001**	**58.011**	**<0.001**
Time*Residence	11,308	**3.607**	**<0.001**	**3.551**	**<0.001**	**12.996**	**<0.001**
Treatment	1,28	**21.396**	**<0.001**	**23.158**	**<0.001**	**54.808**	**<0.001**
Treatment*Residence	1,28	0.035	0.852	1.059	0.312	**6.955**	**0.013**
Time*Treatment	11,308	**9.456**	**<0.001**	**26.476**	**<0.001**	**24.18**	**<0.001**
Time*Treatment* Residence	11,308	0.283	0.989	**4.115**	**<0.001**	**4.003**	**<0.001**

Table S8. Correlations between seedling survival and Beals index in time on different habitat types for species from seed introduction experiment. Gap – seedling survival in gaps, control – seedling survival in the intact vegetation, control/gap – the ratio of seedling survival in the intact vegetation and in gaps. Statistically significant results are in bold.

		Carex acuta-Carex panicea habitat			*Deschampsia cespitosa-Carex tomntosa* habitat			*Sesleria uliginosa-Briza media* habitat		
		r	N	p	r	N	p	r	N	p
Jun-13	gap	**0.465**	**30**	**0.010**	**0.483**	**30**	**0.007**	**0.535**	**30**	**0.002**
	control	**0.531**	**30**	**0.003**	**0.437**	**30**	**0.016**	**0.606**	**30**	**0.000**
	control/gap	**0.629**	**27**	**0.000**	0.139	27	0.488	0.270	27	0.173
Aug-13	gap	**0.462**	**30**	**0.010**	**0.485**	**30**	**0.007**	**0.641**	**30**	**0.000**
	control	**0.516**	**30**	**0.004**	**0.578**	**30**	**0.001**	**0.560**	**30**	**0.001**
	control/gap	**0.645**	**27**	**0.000**	0.330	28	0.086	0.006	27	0.976
Sep-13	gap	**0.460**	**30**	**0.011**	**0.453**	**30**	**0.012**	**0.602**	**30**	**0.000**
	control	**0.460**	**30**	**0.011**	**0.593**	**30**	**0.001**	**0.427**	**30**	**0.019**
	control/gap	**0.451**	**25**	**0.023**	0.315	27	0.109	-0.131	26	0.525
Apr-14	gap	**0.512**	**30**	**0.004**	**0.521**	**30**	**0.003**	**0.664**	**30**	**0.000**
	control	**0.562**	**30**	**0.001**	**0.637**	**30**	**0.000**	**0.672**	**30**	**0.000**
	control/gap	0.353	22	0.107	**0.588**	**24**	**0.003**	-0.134	24	0.533
Jun-14	gap	**0.509**	**30**	**0.004**	**0.519**	**30**	**0.003**	**0.653**	**30**	**0.000**
	control	**0.577**	**30**	**0.001**	**0.644**	**30**	**0.000**	**0.750**	**30**	**0.000**
	control/gap	**0.481**	**22**	**0.023**	**0.570**	**24**	**0.004**	0.320	24	0.128
Aug-14	gap	**0.494**	**30**	**0.006**	**0.457**	**30**	**0.011**	**0.616**	**30**	**0.000**
	control	**0.589**	**30**	**0.001**	**0.603**	**30**	**0.000**	**0.690**	**30**	**0.000**

	control/gap	**0.531**	**22**	**0.011**	**0.598**	**21**	**0.004**	**0.449**	**23**	**0.032**
	gap	**0.499**	**30**	**0.005**	**0.493**	**30**	**0.006**	**0.664**	**30**	**0.000**
Jun-15	control	**0.566**	**30**	**0.001**	**0.606**	**30**	**0.000**	**0.672**	**30**	**0.000**
	control/gap	**0.485**	**21**	**0.026**	**0.593**	**21**	**0.005**	**0.453**	**22**	**0.034**
	gap	**0.479**	**30**	**0.007**	**0.488**	**30**	**0.006**	**0.653**	**30**	**0.000**
Sep-15	control	**0.572**	**30**	**0.001**	**0.603**	**30**	**0.000**	**0.750**	**30**	**0.000**
	control/gap	**0.447**	**21**	**0.042**	**0.595**	**21**	**0.004**	**0.496**	**22**	**0.019**
	gap	**0.416**	**30**	**0.022**	0.357	30	0.053	**0.534**	**30**	**0.002**
Jun-16	control	**0.536**	**30**	**0.002**	**0.385**	**30**	**0.036**	**0.649**	**30**	**0.000**
	control/gap	**0.468**	**21**	**0.032**	0.369	20	0.109	**0.445**	**21**	**0.043**
	gap	**0.402**	**30**	**0.027**	0.358	30	0.052	**0.530**	**30**	**0.003**
Sep-16	control	**0.538**	**30**	**0.002**	**0.385**	**30**	**0.036**	**0.649**	**30**	**0.000**
	control/gap	**0.486**	**21**	**0.025**	0.369	20	0.110	**0.458**	**21**	**0.037**
	gap	0.285	30	0.126	0.257	30	0.171	0.298	30	0.110
Aug-17	control	**0.466**	**30**	**0.009**	0.235	30	0.210	**0.593**	**30**	**0.001**
	control/gap	0.439	16	0.089	0.523	14	0.055	**0.667**	**13**	**0.013**
	gap	0.189	30	0.318	0.283	30	0.129	0.232	30	0.217
Jul-18	control	**0.405**	**30**	**0.026**	0.192	30	0.310	**0.455**	**30**	**0.012**
	control/gap	0.507	15	0.054	0.535	11	0.090	0.517	10	0.126

Table S9. Correlations between seedling survival in time on different habitat types (in pairs) for species from seed introduction experiment. Gap – seedling survival in gaps, control – seedling survival in the intact vegetation, control/gap – the ratio of seedling survival in the intact vegetation and in gaps. Statistically significant results are in bold.

		Carex acuta-Carex panicea / Deschampsia cespitosa-Carex tomntosa habitat			*Carex acuta-Carex panicea / Sesleria uliginosa-Briza media* habitat			*Deschampsia cespitosa-Carex tomntosa / Sesleria uliginosa-Briza media* habitat		
		r	N	p	r	N	p	r	N	p
Jun-13	gap	**0.799**	**30**	**0.000**	**0.832**	**30**	**0.000**	**0.572**	**30**	**0.001**
	control	**0.849**	**30**	**0.000**	**0.838**	**30**	**0.000**	**0.679**	**30**	**0.000**
	control/gap	**0.531**	**26**	**0.005**	**0.492**	**26**	**0.011**	0.132	27	0.511
Aug-13	gap	**0.754**	**30**	**0.000**	**0.737**	**30**	**0.000**	**0.592**	**30**	**0.001**
	control	**0.810**	**30**	**0.000**	**0.739**	**30**	**0.000**	**0.676**	**30**	**0.000**
	control/gap	**0.588**	**27**	**0.001**	0.159	26	0.439	-0.002	27	0.992
Sep-13	gap	**0.701**	**30**	**0.000**	**0.668**	**30**	**0.000**	**0.500**	**30**	**0.005**
	control	**0.681**	**30**	**0.000**	**0.533**	**30**	**0.002**	**0.502**	**30**	**0.005**
	control/gap	**0.574**	**24**	**0.003**	0.081	23	0.713	-0.152	25	0.470
Apr-14	gap	**0.895**	**30**	**0.000**	**0.782**	**30**	**0.000**	**0.618**	**30**	**0.000**
	control	**0.735**	**30**	**0.000**	**0.880**	**30**	**0.000**	**0.709**	**30**	**0.000**
	control/gap	0.376	21	0.093	0.018	21	0.937	-0.041	22	0.856
Jun-14	gap	**0.913**	**30**	**0.000**	**0.796**	**30**	**0.000**	**0.637**	**30**	**0.000**
	control	**0.757**	**30**	**0.000**	**0.897**	**30**	**0.000**	**0.822**	**30**	**0.000**
	control/gap	**0.437**	**21**	**0.047**	**0.579**	**21**	**0.006**	**0.520**	**22**	**0.013**
Aug-14	gap	**0.918**	**30**	**0.000**	**0.743**	**30**	**0.000**	**0.587**	**30**	**0.001**

	control	**0.792**	**30**	**0.000**	**0.939**	**30**	**0.000**	**0.723**	**30**	**0.000**
	control/gap	0.372	20	0.106	**0.893**	**20**	**0.000**	0.082	20	0.731
	gap	**0.942**	**30**	**0.000**	**0.692**	**30**	**0.000**	**0.581**	**30**	**0.001**
Jun-15	control	**0.835**	**30**	**0.000**	**0.816**	**30**	**0.000**	**0.664**	**30**	**0.000**
	control/gap	**0.530**	**20**	**0.016**	**0.768**	**20**	**0.000**	0.247	20	0.294
	gap	**0.948**	**30**	**0.000**	**0.677**	**30**	**0.000**	**0.581**	**30**	**0.001**
Sep-15	control	**0.895**	**30**	**0.000**	**0.840**	**30**	**0.000**	**0.725**	**30**	**0.000**
	control/gap	**0.605**	**20**	**0.005**	**0.870**	**20**	**0.000**	**0.528**	**20**	**0.017**
	gap	**0.938**	**30**	**0.000**	**0.671**	**30**	**0.000**	**0.553**	**30**	**0.002**
Jun-16	control	**0.362**	**30**	**0.050**	**0.854**	**30**	**0.000**	0.145	30	0.444
	control/gap	0.345	20	0.137	**0.877**	**19**	**0.000**	0.227	18	0.365
	gap	**0.946**	**30**	**0.000**	**0.665**	**30**	**0.000**	**0.570**	**30**	**0.001**
Sep-16	control	**0.388**	**30**	**0.034**	**0.841**	**30**	**0.000**	0.145	30	0.444
	control/gap	0.341	20	0.141	**0.874**	**19**	**0.000**	0.229	18	0.362
	gap	**0.865**	**30**	**0.000**	**0.650**	**30**	**0.000**	**0.423**	**30**	**0.020**
Aug-17	control	**0.445**	**30**	**0.014**	0.361	30	0.050	-0.021	30	0.914
	control/gap	0.238	13	0.434	**0.617**	**12**	**0.033**	**0.852**	**10**	**0.002**
	gap	**0.807**	**30**	**0.000**	**0.747**	**30**	**0.000**	**0.638**	**30**	**0.000**
Jul-18	control	**0.850**	**30**	**0.000**	**0.510**	**30**	**0.004**	-0.019	30	0.923
	control/gap	0.055	10	0.880	0.522	10	0.122	**0.826**	**8**	**0.011**

Table S10: Repeated Measurement ANOVA of transplant survival of resident and non-resident species (“habitat residence”) in gaps and control plots (Treatment) during the experiment in different habitat types (separate analysis for each habitat type). Statistically significant results are in bold.

	Carex acuta-Carex panicea habitat			*Deschampsia caespitosa-Carex tomentosa habitat*			*Sesleria uliginosa-Briza media habitat*		
	Degree of freedom	F	p	Degree of freedom	F	p	Degree of freedom	F	p
Residence	1,18	2.54	0.128	1,22	0.329	0.572	1,22	0.783	0.386
Time	**10,180**	**82.376**	**<0.001**	**10,220**	**31.492**	**<0.001**	**10,220**	**76.332**	**<0.001**
Time*Residence	**10,180**	**2.012**	**0.034**	10,220	1.184	0.303	10,220	0.413	0.939
Treatment	1,18	3.005	0.1	**1,22**	**6.978**	**0.015**	1,22	1.084	0.309
Treatment*Residence	1,18	1.062	0.316	1,22	0.006	0.941	1,22	0.439	0.514
Time*Treatment	**10,180**	**2.162**	**0.022**	**10,220**	**2.562**	**0.006**	10,220	1.43	0.168
Time*Treatment* Residence	10,180	1.806	0.062	10,220	1.29	0.237	10,220	1.763	0.069

Table S11. Correlations between transplant survival and Beals index in time on different habitat types for species from transplanting experiment. Gap – transplant survival in gaps, control – transplant survival in the intact vegetation, control/gap – the ratio of transplant survival in the intact vegetation and in gaps. Statistically significant results are in bold.

		Carex acuta-Carex panicea habitat			*Deschampsia cespitosa-Carex tomentosa* habitat			*Sesleria uliginosa-Briza media* habitat		
		r	N	p	r	N	p	r	N	p
Aug-13	gap	0.348	20	0.133	**0.407**	**24**	**0.048**	0.346	24	0.098
	control	0.297	20	0.203	**0.519**	**24**	**0.009**	0.280	24	0.185
	control/gap	-0.160	20	0.499	-0.127	22	0.573	-0.081	24	0.708
Sep-13	gap	0.402	20	0.079	0.342	24	0.102	0.265	24	0.211
	control	0.413	20	0.070	**0.472**	**23**	**0.023**	0.210	24	0.325
	control/gap	-0.227	20	0.336	-0.122	19	0.618	0.000	24	0.998
Apr-14	gap	**0.492**	**20**	**0.028**	**0.421**	**24**	**0.041**	0.390	24	0.060
	control	**0.486**	**20**	**0.030**	**0.445**	**24**	**0.029**	0.350	24	0.094
	control/gap	0.349	17	0.169	0.155	16	0.566	0.169	22	0.453
Jun-14	gap	**0.528**	**20**	**0.017**	**0.436**	**24**	**0.033**	0.373	24	0.072
	control	**0.686**	**20**	**0.001**	**0.474**	**24**	**0.019**	0.337	24	0.108
	control/gap	0.401	17	0.111	0.306	16	0.249	0.179	22	0.426
Aug-14	gap	**0.522**	**20**	**0.018**	**0.482**	**24**	**0.017**	**0.407**	**24**	**0.049**
	control	**0.686**	**20**	**0.001**	**0.526**	**24**	**0.008**	0.338	24	0.106
	control/gap	0.366	16	0.163	0.387	16	0.139	0.216	21	0.347
Jun-15	gap	**0.492**	**20**	**0.028**	**0.549**	**24**	**0.005**	0.389	24	0.061
	control	**0.759**	**20**	**0.000**	**0.608**	**24**	**0.002**	**0.558**	**24**	**0.005**

	control/gap	**0.722**	**16**	**0.002**	0.397	15	0.143	0.334	17	0.190
	gap	**0.491**	**20**	**0.028**	**0.591**	**24**	**0.002**	0.359	24	0.085
Sep-15	control	**0.759**	**20**	**0.000**	**0.608**	**24**	**0.002**	**0.480**	**24**	**0.018**
	control/gap	**0.734**	**17**	**0.001**	0.307	15	0.266	0.330	17	0.196
	gap	0.438	20	0.053	**0.486**	**24**	**0.016**	0.253	24	0.233
Jun-16	control	**0.746**	**20**	**0.000**	**0.548**	**24**	**0.006**	0.397	24	0.055
	control/gap	**0.706**	**16**	**0.002**	0.418	15	0.121	0.205	16	0.447
	gap	**0.459**	**20**	**0.042**	**0.486**	**24**	**0.016**	0.237	24	0.264
Sep-16	control	**0.778**	**20**	**0.000**	**0.616**	**24**	**0.001**	0.316	24	0.132
	control/gap	**0.763**	**16**	**0.001**	0.487	15	0.065	0.012	18	0.962
	gap	0.233	20	0.323	0.119	24	0.581	0.347	24	0.097
Aug-17	control	**0.768**	**20**	**0.000**	0.270	24	0.203	0.016	24	0.941
	control/gap	0.447	11	0.168	0.461	10	0.180	-0.343	9	0.367
	gap	0.234	20	0.321	0.114	24	0.596	0.208	24	0.330
Jul-18	control	**0.618**	**20**	**0.004**	0.360	24	0.084	0.218	24	0.305
	control/gap	0.514	11	0.106	0.694	8	0.056	-0.139	6	0.793
